# Surface TREM2 on circulating M-MDSCs as a novel prognostic factor for adults with treatment-naïve diffuse large B-cell lymphoma

**DOI:** 10.1186/s40164-023-00399-x

**Published:** 2023-04-07

**Authors:** Hao-Yuan Wang, Fu-Chen Yang, Ching-Fen Yang, Yao-Chung Liu, Po-Shen Ko, Chien-Jung Li, Chun-Kuang Tsai, Yi-Lin Chung, Nien-Jung Chen

**Affiliations:** 1 Division of Hematology and Oncology, Department of Medicine, Taipei Veterans General Hospital, Taipei, Taiwan; 2 Faculty of Medicine, School of Medicine, National Yang Ming Chiao Tung University, Taipei, Taiwan; 3 Program in Molecular Medicine, School of Life Sciences, National Yang Ming Chiao Tung University, Taipei, Taiwan; 4 Institute of Microbiology and Immunology, School of Life Sciences, National Yang Ming Chiao Tung University, Taipei, Taiwan; 5 Department of Pathology and Laboratory Medicine, Taipei Veterans General Hospital, Taipei, Taiwan; 6 Institute of Genome Sciences, School of Life Sciences, National Yang Ming Chiao Tung University, Taipei, Taiwan

**Keywords:** Arginase 1, Cytotoxic T lymphocytes (CTLs), Diffuse large B-cell lymphoma (DLBCL), Monocytic myeloid-derived suppressor cells (M-MDSCs), Triggering receptors expressed on myeloid cells 2 (TREM2)

## Abstract

**Introduction:**

Circulating monocytic myeloid-derived suppressive cells (M-MDSCs) are implicated as a poor prognostic factor and cause CAR T-cell failure in diffuse large B-cell lymphoma (DLBCL). Triggering receptors expressed on myeloid cells 2 (TREM2) are a transmembrane glycoprotein that polarize macrophages to anti-inflammation phenotype but have never been explored on M-MDSCs. This study aims to elucidate the expression and clinical impact of surface TREM2 on circulating M-MDSCs derived from DLBCL adults.

**Methods:**

This prospective, observational study enrolled 100 adults with newly diagnosed and treatment-naïve DLBCL from May 2019 to October 2021. Human circulating M-MDSCs were obtained from freshly isolated peripheral blood, and each patient’s surface-TREM2 level on M-MDSCs was normalized via a healthy control at the same performance of flow-cytometry analysis. Murine MDSCs derived from bone marrow (BM-MDSCs) were adopted to assess the link between Trem2 and cytotoxic T lymphocytes.

**Results:**

More circulating M-MDSCs at diagnosis of DLBCL predicted worse progression-free (PFS) and overall survival (OS). Patients with higher IPI scores, bone marrow involvement, or lower absolute counts of CD4^+^ or CD8^+^ T cells in PB had significantly higher normalized TREM2 levels on M-MDSCs. Additionally, normalized TREM2 levels on M-MDSCs could be grouped into low (< 2%), medium (2–44%), or high (> 44%) levels, and a high normalized TREM2 level on M-MDSCs was proven as an independent prognostic factor for both PFS and OS via multivariate Cox regression analysis and associated with worst PFS and OS. Interestingly, normalized levels of surface TREM2 on M-MDSCs were negatively associated with absolute counts of PB CD8^+^ T cells and positively correlated with levels of intracellular arginase 1 (ARG1) within M-MDSCs. Wild-type BM-MDSCs had significantly higher mRNA levels of *Arg1* and showed more prominent ability to suppress the proliferation of co-cultured CD8^+^ T cells than BM-MDSCs from *Trem2* knockout mice, and the suppressive ability could be impaired by adding Arg1 inhibitors (CB1158) or supplementing L-arginine.

**Conclusion:**

In treatment-naïve DLBCL adults, a high surface-TREM2 level on circulating M-MDSCs is a poor prognostic factor for both PFS and OS and warrants further investigation for its potential as a novel target in immunotherapy.

**Supplementary Information:**

The online version contains supplementary material available at 10.1186/s40164-023-00399-x.

## Key points

- A higher surface-TREM2 level on circulating M-MDSCs is associated with a higher IPI-risk score and worse PFS and OS.
- For circulating M-MDSCs, the surface-TREM2 level is positively correlated to the intracellular-ARG1 level and negatively associated with the absolute count of circulating CD8^+^ T cells.

**Supplementary Information:**

The online version contains supplementary material available at 10.1186/s40164-023-00399-x.

## Introduction

Contemporary immunotherapies, including immune checkpoint inhibitors (ICIs) and chimeric antigen receptor T-cells (CARTs), exert great effort in targeting cytotoxic T lymphocytes (CTLs) and revolutionizing the treatment landscape of many cancer types. However, few patients with diffuse large B-cell lymphoma (DLBCL) have shown clinical benefits from ICIs [1], and the overall response rate was as low as 8% in one recent trial using an anti-PD-1 antibody as monotherapy to treat relapse/refractory DLBCL [2]. Regarding CARTs, recurrence remains a challenging issue, and more than half of DLBCL patients inevitably relapse even when anti-CD19 CARTs are used as a first salvage therapy [3–6].

Recruitment of immunosuppressive cells into a tumor microenvironment is an important mechanism of resistance to immunotherapy targeting CTLs. Recent studies have spotlighted myeloid-derived suppressive cells (MDSCs) as one of the culprits in impairing the function of CTLs and attenuating the effectiveness of immunotherapies [7–11]; inhibition of trafficking MDSCs into tumor sites could enhance the efficacy of immunotherapies [12].

In fact, MDSCs represent a collection of myeloid-lineage immature cells boasting various immunosuppressive abilities to facilitate tumor growth, such as reprogramming macrophages into M2-like phenotypes, driving T lymphocyte differentiation into Treg, depleting essential metabolites for T lymphocyte fitness, and producing reactive nitrogen species to alter the correct constructure between neoantigen peptides and MHC I, and affecting TCR recognition by T lymphocytes [13, 14].

MDSCs can be phenotyped into two major groups: monocytic MDSCs (M-MDSCs) and polymorphonucler MDSCs (PMN-MDSCs). Earlier studies have demonstrated that a higher percentage of circulating M-MDSCs in peripheral blood (PB) is associated with more advanced stages [15], higher IPI risk [16], and worse survival [15, 17] in DLBCL patients.

Triggering receptors expressed on myeloid cells 2 (TREM2) are transmembrane glycoproteins expressed on myeloid-lineage cells, such as dendritic cells, monocytes, and macrophages [18]. TREM2 have been implicated in promoting the survival of macrophages [19] and are well known for polarizing macrophages to M2-like phenotypes presenting anti-inflammation properties [20–22]. Despite abundant evidence regarding TREM2 on macrophages, currently there are no published studies that address the expression and roles of TREM2 on M-MDSCs.

Given that (1) M-MDSCs possess immunosuppressive ability and impair immunotherapeutic effectiveness in DLBCL patients, (2) a higher percentage of circulating M-MDSCs in PB is associated with worse survival in DLBCL patients, (3) both M-MDSCs and M2 macrophages belong to the monocytic lineage, and (4) TREM2 are well known M2-polarization markers and exert an anti-inflammatory nature, we hypothesize that TREM2 could be expressed on M-MDSCs to augment their immunosuppressive ability, leading to a poor outcome in DLBCL patients.

This prospective and observational study aims to answer whether TREM2 are expressed on M-MDSCs cell surface and attempts to elucidate the clinical impact of surface TREM2 on circulating M-MDSCs derived from patients with newly diagnosed and treatment-naïve DLBCL.

## Materials and methods

### Human patients

This was a prospective and observational study conducted in a tertiary medical center in Taiwan. Adult patients with newly diagnosed and treatment-naïve DLBCL were eligible for enrollment from May 15, 2019 to October 31, 2021, in the Division of Hematology and Oncology at Taipei Veterans General Hospital. Patients with DLBCL originating from the central nervous system (primary central nervous system lymphoma) or mediastinum were excluded because their frontline treatments were obviously different from DLBCL stemming from other origins. Additionally, patients with human immunodeficiency virus infection and other concurrent malignancies were excluded.

This study was approved by the Institutional Review Board of Taipei Veterans General Hospital (no. 2019-04-015CC, 2020-07-024BCF and 2022-01-031CC), and written informed consent was obtained from each enrolled patient with respect to the use of their blood for scientific purposes.

International Prognostic Index (IPI) scores were calculated using five independent risk factors, including (1) age > 60, (2) performance status > 1, (3) elevated serum lactate dehydrogenase, (4) number of extra-nodal sites of disease > 1, and (5) Ann Arbor stage III or IV. Three prognostic risk categories were developed based on the above five risk factors: low risk, 0 to 1 factor; intermediate risk, 2 to 3 factors; high risk, 4 to 5 factors [23].

The cell of origin (COO) of GCB (germinal center B-cell) and non-GCB subtypes were determined using the Hans algorithm, utilizing immunohistochemical (IHC) staining of CD10, BCL6, and MUM1 [24]. Double-expressor lymphoma (DEL) is defined as the expression of MYC protein (≧ 40% of tumor cells) and BCL2 protein (≧ 50% of tumor cells) by IHC staining [25]. Double-hit lymphoma (DHL) is defined as concurrent rearrangements of MYC and BCL2 and/or BCL6 by fluorescence in situ hybridization (FISH) analysis [26].

### Detecting M-MDSCs and analyzing TREM2 levels via flow-cytometry analysis

Human circulating M-MDSCs (defined as CD15^−^ CD14^+^ CD11b^+^ CD33^+^ HLA-DR^low/−^ cells) [27, 28] were obtained from freshly isolated peripheral-blood white cells (PBWCs) before treatment initiation (including steroids). The TREM2 levels on the M-MDSC surface were analyzed using staining with a combination of fluorescent-labeled monoclonal antibodies: rat anti-human TREM2 (clone # 237920; cat no. FAB17291p; R&D system), mouse anti-human CD45 (clone HI30; cat no. 304035; Biolegend), mouse anti-human CD15 (clone MC-480; cat no. 125613; Biolegend), mouse anti-human CD14 (clone M5E2; cat no. 555397; BD Bioscience), rat anti-human CD11b (clone M1/70; cat no. 101225; Biolegend), mouse anti-human CD33 (clone P67.6; cat no. 366623; Biolegend), and mouse anti-human HLA-DR (clone L243; cat no. 307639; Biolegend). Dead cells were excluded from the analysis by staining with propidium iodide (Sigma Aldrich).

Cells were acquired using a FACSFortessa instrument (BD Biosciences) and analyzed using the FlowJo cytometric analysis program (Tree Star). To increase inter-experiment comparability, PB from the principal investigator (Hao-Yuan Wang) was used as an internal control (Healthy Control, Additional file 1: Figure S1A), and every patient’s blood sample was paired to the healthy control at the same performance of flow-cytometry analysis to calculate the normalized MFI change of TREM2, as follows: (patient’s TREM2 MFI - healthy control’s TREM2 MFI)/(healthy control’s TREM2 MFI) × 100%.

### Analyzing levels of arginase 1 within M-MDSCs

Analysis of the intracellular arginase 1 (ARG1) level within M-MDSCs was performed on fresh PBWCs from the latest 31 DLBCL patients. PBWCs were initially stained with fluorescent-labeled monoclonal antibodies against cell-surface markers: TREM2, CD14, CD11b, CD33, HLA-DR, CD15, and CD45 (as described above). Cells were then fixed with Cytofix/Cytoperm and permeabilized with a perm/wash buffer (Thermo Fisher Scientific, cat no. 1952895 and 2270788), according to the manufacturer’s protocol. Finally, cells were intracellularly stained (30 min. at RT) with mouse anti-human ARG1 (clone 14D2C43; cat no. 369705; Biolegend). Fluorescence minus one (FMO) control was used for the gating.

The PB from the principal investigator (Hao-Yuan Wang) was used as an internal control (healthy control, Additional file 1: Figure S1B). The normalized MFI change of ARG1 is defined as follows: (patient’s ARG1 MFI - healthy control’s ARG1 MFI)/(healthy control’s ARG1 MFI) × 100%.

### Quantifying CD4^+^ T cells and CD8^+^ T cells in PB

Freshly isolated PBWCs were stained with Beckman Coulter CYTO-STAT tetraCHROME monoclonal antibodies: (1) CD45, CD3, CD4, and CD8 (cat no. 6607013), and (2) CD45, CD3, CD19, and CD56 (cat no. 6607073). Flow-cytometric enumeration of CD4^+^ T cells and CD8^+^ T cells was done for all enrolled patients with a NAVIOS^™^ flow cytometer (BECKMAN) and analyzed using Navios Tetra software. Total white blood cell counts (WBCs) and absolute lymphocyte counts (ALCs) were determined on a Coulter XN-20 (SYSMEX) machine, and then absolute values of CD4^+^ T cells and CD8^+^ T cells were calculated by multiplying the subject’s ALC by the percentage of CD4^+^ T cells and CD8^+^ T cells subset obtained by flow cytometry, respectively.

### RNA extraction and real-time PCR analysis

RNA was extracted using an RNA purification kit (Qiagen), following the manufacturer’s instructions. Complementary DNA (cDNA) was generated using a high-capacity cDNA reverse transcription kit (Applied Biosystems). Quantitative real-time PCR analysis was performed on an Mx3000P^™^ instrument (Strategene), using the KAPA SYBR FAST qPCR master mix (Kapa Biosystems) and running for 40 cycles of universal cycling conditions (95 °C for 10 min, followed by 40 cycles at 95 °C for 30 s, 60 °C for 30 s, and 72 °C for 30 s). Analysis utilized the cycle quantification value (Cq), and presented as 2^−ΔCq^; ΔCq was the difference between the Cq of the target gene (Cqt) and the reference gene (Cqr) (ΔCq = Cqt−Cqr). Patients’ data were initially normalized to each participant’s GAPDH transcriptional level (endogenous control) and the healthy donor’s mean GAPDH transcriptional level.

Primers were shown as follows: hTREM2, 5′-CTCTTTGTCACAGAGCTGTC-3′; hARG1, 5′-GGCAAGGTGATGGAAGAAAC-3′; mTrem2, 5′-GTCCTGACTGTTGCTCAATC-3′; mArg1, 5′-GGGTGGAGACCACAGTCTG-3′.

### Mice

Conventional *Trem2* knockout (KO) mice of the C57BL/6 genetic background were originally established in T. W. Mak's laboratory (Toronto, Ontario, Canada) and maintained under specific pathogen-free conditions (but positive for *Helicobacter spp.*) in the animal center at National Yang Ming Chiao Tung University in accordance with Institutional Animal Care and Use Committee guidelines (1080916r). Age-matched males (8–12 weeks old) were used for experiments.

### Differentiation of bone marrow–derived myeloid-derived suppress cells (BM-MDSCs)

Total bone marrow (BM) cells were isolated from wild-type (WT) or *Trem2* KO mice and induced to differentiate into MDSCs as previously described [29, 30]. Briefly, 1.5 million bone marrow cells were plated on a 6-well culture dish and incubated for 6 days with 40 ng/ml of GMCSF and 40 ng/ml of IL-4 in RPMI medium 1640 (Thermo Fisher Scientific), containing 10% fetal bovine serum (FBS; Gibco) and 1% antibiotics (100 mg/ml penicillin/streptomycin; Biological Industries). Adherent cells were deemed to be BM-MDSCs.

### MDSC-induced suppression of T-cell proliferation

Splenic cells were isolated by mashing spleen through a 40 μm Nylon strainer (BD Biosciences). Red blood cells were removed using an ACK lysis buffer (150 mM NH_4_Cl, 1 mM KHCO_3_, 0.1 mM Na_2_EDTA). Single-cell suspensions of splenic cells were resuspended in PBS contained 5 μM CFSE for 20 min incubation at 37 °C. After labeling, CFSE-labeled splenic cells were plated on an anti-CD3/CD28 pre-coated 24- or 96-well culture dish, and co-cultured with 12% of BM-MDSCs for 48 h. For complementation experiments, L-arginine (300 nM; Sigma-Aldrich) or CB1158 (5 μM; BioVision) were supplied in the co-culture system. After 48 h, co-cultured splenic cells were harvested and stained with antibodies to CD8 (53–6.7; eBioscience) antibody, and were acquired on a FACSFortessa instrument (BD Biosciences).

### Statistical analysis

For human data, Mann–Whitney *U* tests were used for quantitative data, Fisher exact tests for categorical data, and the Kaplan–Meier estimate and log-rank test for survival data. Follow-up was continued until death or July 31, 2022. Endpoints for overall survival (OS) and progression-free survival (PFS) were defined as death due to any cause, and disease progression or death, respectively. The cut-off percentage of circulating M-MDSCs and the cut-off levels for normalized MFI changes of surface TREM2 were determined by ROC analysis and AUC to select best one and two cut-points which had maximum sum of sensitivity and specificity values, respectively. The cut-off values of age, absolute counts of CD4^+^ T cells and CD8^+^ T cells in PB were determined by their medians. Cox regression models were used to calculate hazard ratios (HRs), and variables must fulfil the proportional hazard assumption; all candidate factors with a *p*-value of < 0.2 in univariate analyses were subsequently entered into a multivariate regression model. The HRs of all factors were reported with the corresponding *p*-values and 95% confidence intervals (CIs). Pearson's correlation coefficients were used to evaluate the association between the levels of TREM2 and ARG1. All statistical analyses were performed using IBM SPSS version 22.0 (IBM Corp., Armonk, NY, USA); a two-tailed *p*-value of < 0.05 was considered statistically significant.

Murine data from at least two independent experiments were analyzed using GraphPad Prism software, version 6.0 (GraphPad Software). All results were calculated and expressed as the mean ± SEM, and group mean values were evaluated using the Mann–Whitney nonparametric test or the unpaired t-test with Welch’s correction.

## Results

As shown in Table 1 and Additional file 1 (Figure S2), the study prospectively enrolled 100 adults with treatment-naïve DLBCL, with a median age of 70, 40% of patients being stage IV, 33% showing a high-risk IPI score, 26% having bone marrow (BM) involvement, 26% being the GCB type according to Hans algorithm by IHC stains, 41% having double-expressor lymphoma, and the median absolute count of CD4^+^ T cells and CD8^+^ T cells in PB being 506 and 390/µL, respectively.

**Table 1:** Characteristics of 100 patients with treatment-naïve diffuse large B-cell lymphoma

Number of patients (%)	Number of patients (%). Median of value [interquartile-range]
Age, years	70 [60–79]
> 70	50 (50%)
≤ 70	50 (50%)
Sex	
Male	64 (64%)
Hepatitis B carrier^a^	14 (14%)
Ann-Arbor stage	
I	19 (19%)
II	21 (21%)
III	20 (20%)
IV	40 (40%)
IPI score	
0	6 (6%)
1	19 (19%)
2	16 (16%)
3	26 (26%)
4	19 (19%)
5	14 (14%)
BM involvement	26 (26%)
Complex karyotype	9 (9%)
Bulky mass > 7.5 cm	36 (36%)
Hemophagocytic lymphohistiocytosis^b^	3 (3%)
Cell of origin by IHC stain^c^	
GCB type^d^	26 (26%)
Non-GCB type	74 (74%)
Double-expressor lymphoma^e^	41 (41%)
Epstein-Barr virus-positive diffuse large B-cell origin	6 (6%)
Transformation from previous indolent lymphoma^f^	5 (5%)
T cells in PB	
CD4^+^ T cells (absolute count/µL)	506 [278–772]
CD8^+^ T cells (absolute count/µL)	390 [269–529]
Ratio of CD4^+^/CD8^+^ T cells	1.38 [0.84–2.08]
Percentage of M-MDSC among CD45^+^ cells in PB	0.96% [0.38–1.95]
Frontline treatment	
Induction immunochemotherapy followed by HDC/ASCT	17 (17%)
Induction immunochemotherapy only	71 (71%)
Failed or incomplete induction	12 (12%)
Induction regimen of immunochemotherapy	
R & CHOP-like	76 (76%)
R & EPOCH	19 (19%)
R & Hyper-CVAD	2 (2%)
Other^g^	3 (3%)
Treatment response after frontline treatment	
CR	82 (82%)
PR	6 (6%)
SD/PD/Death^h^	0/7/5 (0/7/5%)

^a^Fourteen patients with positive HBs antigen; 78 patients with positive anti-HBc IgG antibody

^b^Defined according to the HLH-2004 criteria

^c^Defined according to the Hans algorithm

^d^Examined all 26 GCB-type DLBCL patients for FISH of *MYC*, *BCL2*, and *BCL6*: six patients diagnosed as double-hit lymphoma, one patient undetermined due to severely crushing samples

^e^Defined as the expression of MYC and BCL2 in lymphoma cells ≧40% and ≧50%, respectively

^f^Two patients from follicular lymphoma; two patients from marginal zone lymphoma; one patient from mature B cell neoplasm

^g^Three patients died before initiating immunochemotherapy: two received rituximab, and one received merely steroid

^h^Five patients died before response evaluation

BM, bone marrow; CR, complete remission; GCB, germinal center B cell; HDC/ASCT, high-dose chemotherapy and autologous stem cell transplantation; IHC, immunohistochemistry; IPI, international prognostic index; M-MDSCs, monocytic myeloid-derived suppressor cells; PB, peripheral blood; PD, progressive disease; PR, partial remission; R, rituximab; SD, stable disease

After a median follow-up of 548 days (18 months), both the median PFS and OS were non-reach (Additional file 1: Figure S3). R-CHOP-like (rituximab, cyclophosphamide, doxorubicin, vincristine, steroid) was the most common regimen of 1^st^ line immunochemotherapy, 17% of patients received frontline high-dose chemotherapy with autologous stem-cell transplantation, and the complete remission rate after frontline treatment was 82% (Additional file 1: Figure S4).

### Higher percentage of circulating M-MDSCs among PBWCs associated with worse prognosis

**Fig. 1:**
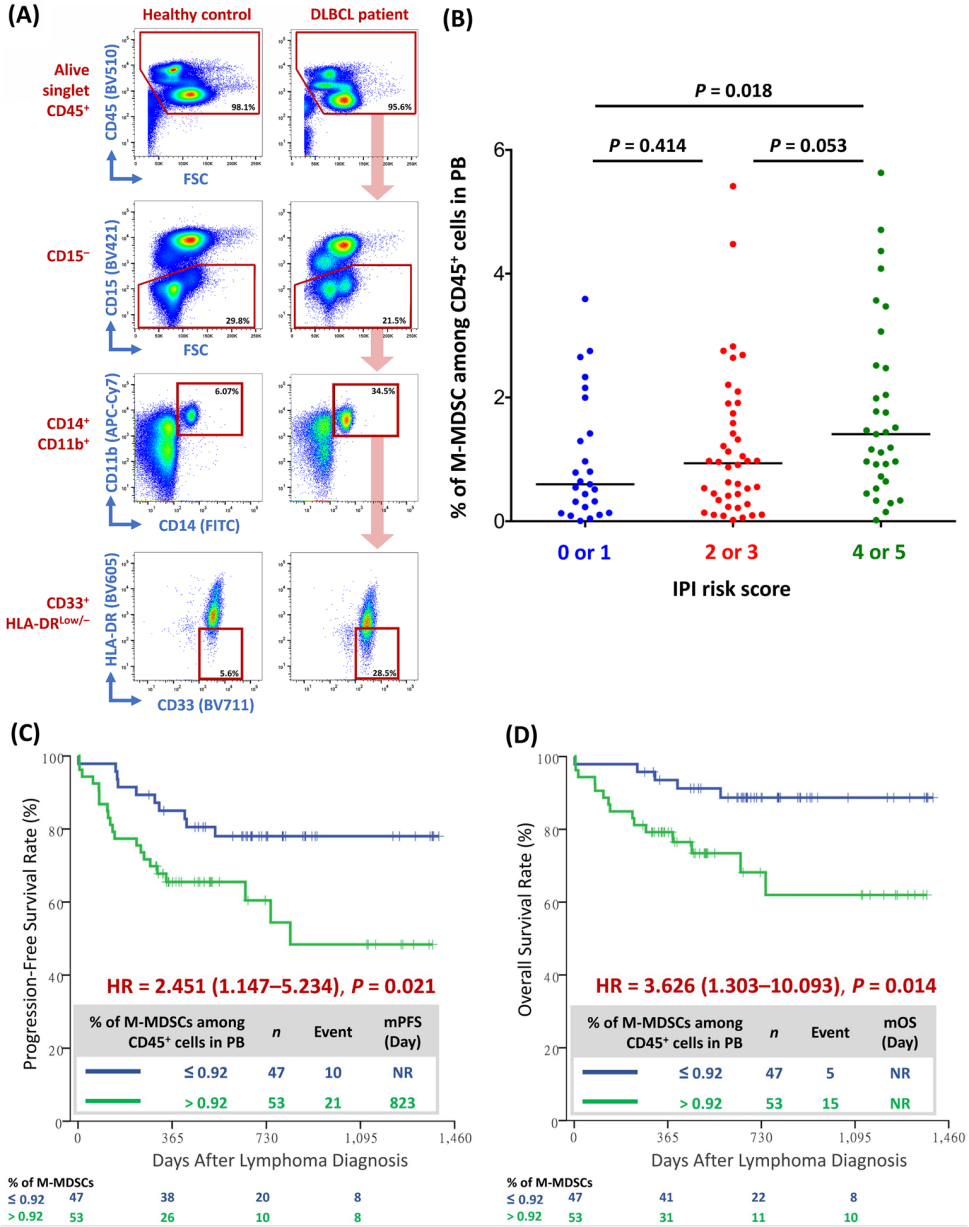
Clinical impact of circulating M-MDSCs on DLBCL patients. **A** Gating strategy of circulating M-MDSCs derived from healthy control’s or DLBCL patient’s PB using flow-cytometry analysis. **B** Percentage of circulating M-MDSCs among PB CD45^+^ cells in DLBCL patients with low, intermediate, or high-risk IPI scores. **C** Progression-free survival and **D** overall survival in DLBCL patients with low or high percentage of circulating M-MDSCs among PB CD45^+^ cells; the cut-off percentage of circulating M-MDSCs as 0.92% determined by ROC analysis and AUC. FSC, forward scatter; HR, hazard ratio; IPI, international prognostic index; M-MDSCs, monocytic myeloid-derived suppressor cells; mOS, median overall survival; mPFS, median progression-free survival; *n*, number; NR, non-reach; PB, peripheral blood

Our study indicates a strong association between circulating M-MDSCs (CD45^+^ CD15^−^ CD14^+^ CD11b^+^ CD33^+^ HLA-DR^low/−^ cells, Fig. 1A) and clinical prognosis in DLBCL patients. Patients with higher IPI scores (Fig. 1B) had significantly higher percentages of M-MDSCs among PBWCs. Regarding patient survival, those with higher percentages of M-MDSCs among PBWCs had significantly worse PFS (Fig. 1C) and OS (Fig. 1D).

### TREM2 expressed on cell surface of M-MDSCs

**Fig. 2:**
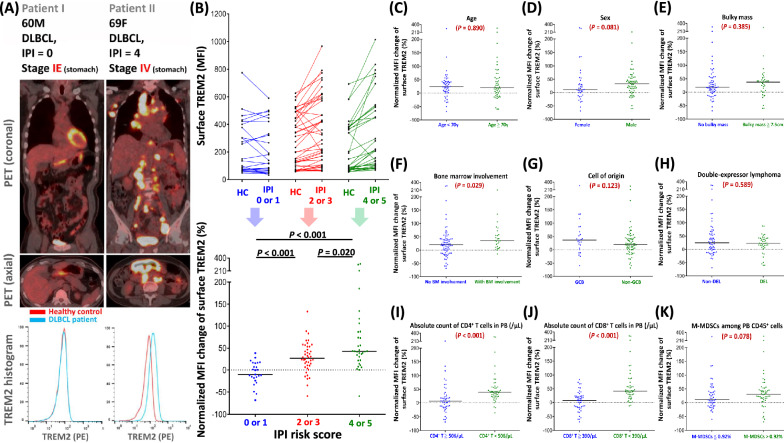
Surface-TREM2 level on M-MDSCs from DLBCL patients. **A** Clinical characteristics (age, sex, IPI score, and Ann Arbor stage), representative coronal and axial view of PET image, and histogram of TREM2 level on PB M-MDSCs in two DLBCL patients. **B** Normalized MFI changes of surface TREM2 on PB M-MDSCs being subcategorized according to IPI risk scores; each line in the upper panel representing a pair of patient samples and the healthy control; each dot in the lower panel being calculated as follows: (patient’s TREM2 MFI—healthy control’s TREM2 MFI)/(healthy control’s TREM2 MFI) × 100%. Normalized MFI changes of surface TREM2 on PB M-MDSCs being subcategorized by **C** age, **D** sex, **E** bulky mass, **F** bone marrow involvement, **G** cell of origin,** H** double-expressor lymphoma, **I** absolute count of CD4^+^ T cells in PB, **J** absolute count of CD8^+^ T cells in PB, or **K** percentage of circulating M-MDSCs among PB CD45.^+^ cells. BM, bone marrow; DEL, double-expressor lymphoma; DLBCL, diffuse large B-cell lymphoma; GCB, germinal center B cell; HC, healthy control; IPI, international prognostic index; M-MDSCs, monocytic myeloid-derived suppressor cells; MFI, mean fluorescence intensity; PB, peripheral blood; PET, positron emission tomography; TREM2, triggering receptors expressed on myeloid cells 2

As illustrated in Fig. 2A, we discovered that TREM2 were expressed on the cell surface of M-MDSCs. Patient II had advanced-stage DLBCL and a high IPI score, expressing higher surface TREM2 on M-MDSCs when compared to the healthy control’s TREM2; in contrast, the level of surface TREM2 on M-MDSCs from patient I, with limited-stage DLBCL and a low IPI score, was similar to those from the healthy control.

### Higher TREM2 level on M-MDSC cell surface associated with higher IPI score and lower absolute count of CD8^+^ T cells

After pooling all normalized TREM2 values from 100 DLBCL patients, the levels of surface TREM2 on M-MDSCs from high-risk DLBCL patients were significantly higher than those from patients with low-risk IPI scores (Fig. 2B).

The normalized MFI changes of surface TREM2 were also significantly higher in DLBCL patients with BM involvement (Fig. 2F), low absolute count of CD4^+^ T cells in PB (Fig. 2I), or low absolute count of CD8^+^ T cells in PB (Fig. 2J). Additionally, patients with a higher percentage of circulating M-MDSCs were noticed to have a trend of higher surface-TREM2 levels (Fig. 2K).

### Higher surface-TREM2 level on M-MDSCs associated with worse prognosis

**Fig. 3:**
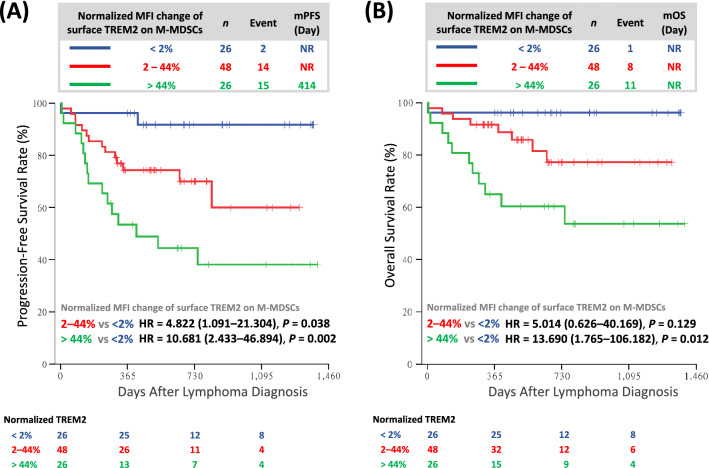
Clinical impact of surface TREM2 on M-MDSCs on DLBCL patients. **A** Progression-free survival and **B** overall survival in DLBCL patients with low, medium, or high normalized levels of surface TREM2 on M-MDSCs. Normalized MFI changes of surface TREM2 on M-MDSCs being grouped into < 2%, 2–44%, and > 44%; cut-off values for normalized MFI changes of surface TREM2 determined by ROC analysis and AUC. HR, hazard ratio; M-MDSCs, monocytic myeloid-derived suppressor cells; MFI, mean fluorescence intensity; mOS, median overall survival; mPFS, median progression-free survival; *n*, number; NR, non-reach; TREM2, triggering receptors expressed on myeloid cells 2

The normalized MFI changes of surface TREM2 could be subcategorized into three groups: low level (< 2%), medium level (2–44%), and high level (> 44%). Importantly, patients whose circulating M-MDSCs expressed higher normalized MFI change of surface TREM2 had significantly worse PFS and OS than those with lower normalized MFI change of surface TREM2 (Fig. 3A, B).

Besides the higher percentage of circulating M-MDSCs among PBWCs and the higher surface-TREM2 level on M-MDSCs, age (more than 70), high-risk IPI score, having BM involvement, low absolute count of CD4^+^ T cells in PB (< 506/µL), and low absolute count of CD8^+^ T cells in PB (< 390/µL) were demonstrated as poor prognostic factors (Additional file 1: Figures S5, S6).

### High surface-TREM2 level on M-MDSCs as an independent predictor of poor survival

To validate the role of surface-TREM2 level on M-MDSCs in predicting survival in adults with treatment-naïve DLBCL, eleven factors (age > 70, being female, IPI risk, bulky mass > 7.5 cm, BM involvement, non-GCB type, double expressor lymphoma, absolute count of CD4^+^ T cells in PB < 506/µL, absolute count of CD8^+^ T cells in PB < 390/µL, the percentage of circulating M-MDSCs among PBWCs > 0.92%, and normalized MFI changes of surface TREM2 on M-MDSCs) were initially verified by univariate analysis and subsequently put into multivariate analysis if qualified, as shown in Table 2. For PFS, age (> 70), being female, having BM involvement, and higher normalized MFI change of surface TREM2 on M-MDSCs were independent predictors of poor outcome. With regard to OS, age (> 70) and high normalized MFI change of surface TREM2 on M-MDSCs (> 44%) were independent predictors of poor survival.

**Table 2:** Univariate and multivariate analyses of surface-TREM2 level on M-MDSCs predicting survival in treatment-naive DLBCL patients

Clinical factors	Progression-free survival				Overall survival			
	Univariate		Multivariate^a,b^		Univariate		Multivariate^a,b^	
	HR (95% CI)	*P*	HR (95% CI)	*P*	HR (95% CI)	*P*	HR (95% CI)	*P*
Age > 70	2.574 (1.210–5.473)	0.014	4.173 (1.777–9.803)	0.001	4.997 (1.667–14.975)	0.004	3.992 (1.309–12.172)	0.015
Female	1.737 (0.858–3.516)	0.125	2.420 (1.162–5.043)	0.018	1.455 (0.603–3.512)	0.404		
IPI risk High risk Intermediate risk Low risk	9.060 (2.089–39.300) 3.989 (0.891–17.849) 1	0.003 0.070			11.362 (1.476–87.444) 4.315 (0.531–35.088) 1	0.020 0.172		
Bulky mass > 7.5 cm	0.972 (0.466–2.031)	0.940	–	–	0.767 (0.295–1.998)	0.587	–	–
BM involvement	3.238 (1.598–6.560)	0.001	2.740 (1.280–5.865)	0.009	1.981 (0.809–4.852)	0.135		
Non-GCB type^**c**^	1.642 (0.673–4.008)	0.276	–	–	1.516 (0.506–4.540)	0.457	–	–
Double expressor lymphoma	1.874 (0.923–3.804)	0.082	2.613 (1.233–5.539)	0.012	1.342 (0.554–3.252)	0.515	–	–
Absolute count of CD4^+^ T cells in peripheral blood < 506/µL	2.208 (1.054–4.624)	0.036			2.083 (0.829–5.233)	0.118		
Absolute count of CD8^+^ T cells in peripheral blood < 390/µL	2.507 (1.179–5.331)	0.017			2.577 (0.989–6.714)	0.053		
Percentage of M-MDSCs among CD45^+^ cells in PB > 0.92%	2.451 (1.147–5.234)	0.021			3.626 (1.303–10.093)	0.014	2.409 (0.843–6.889)	0.101
Normalized MFI change of surface TREM2 on M-MDSCs > 44% 2–44% < 2%	10.681 (2.433–46.894) 4.822 (1.091–21.304) 1	0.002 0.038	8.332 (1.838–37.778) 4.425 (0.980–19.971)	0.006 0.053	13.690 (1.765–106.182) 5.014 (0.626–40.169) 1	0.012 0.129	10.300 (1.307–81.169) 4.217 (0.510–34.834)	0.027 0.182

^a^Multivariate Cox regression model (the backward stepwise method) included all available variables with *P* < 0.200

^b^Age and sex were forced into the multivariate analysis because they may confound between-subject comparisons

^c^According to the Hans algorithm, by immunohistochemistry stain

CI, confidence interval; DLBCL, diffuse large B-cell lymphoma; GCB, germinal center B cell; HR, hazard ratio; IPI, international prognostic index; M-MDSCs, monocytic myeloid-derived suppressor cells; TREM2, triggering receptors expressed on myeloid cells 2; PB, peripheral blood

### Higher surface-TREM2 level on M-MDSCs associated with lower absolute count of CD8^+^ T cells in PB

**Fig. 4:**
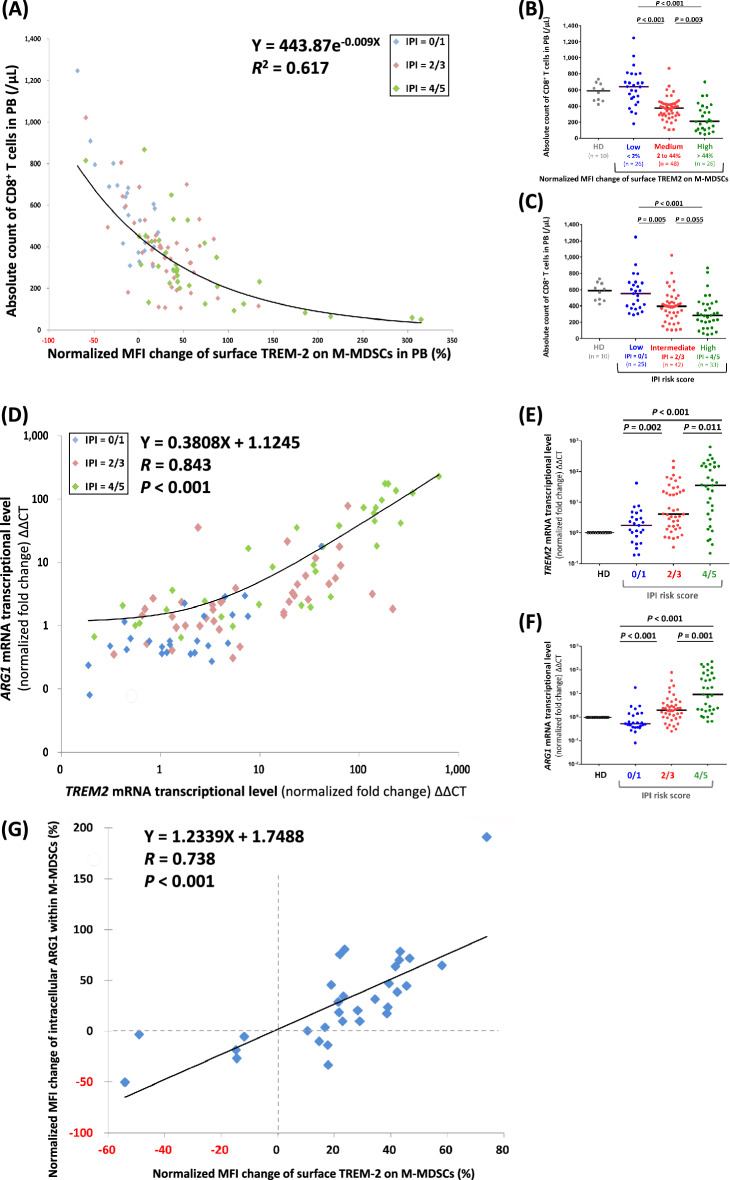
Level of surface TREM2 on M-MDSCs being negatively associated with absolute count of CD8^+^ T cells and positively correlated with level of intracellular ARG1 within M-MDSCs. **A**–**C** The association between normalized MFI changes of surface TREM2 on PB M-MDSCs and the absolute counts of PB CD8^+^ T cells; the absolute counts of CD8.^+^ T cells in PB being subcategorized by ‘‘low, medium, and high normalized TREM2 levels’’ or “low, intermediate, and high IPI risk scores.” **D**–**F** For PB white cells derived from all 100 DLBCL patients, the correlation between the mRNA transcriptional level of *TREM2* and *ARG1* being calculated by ΔΔCT (Pearson’s *R* value = 0.843, *P* < 0.001); mRNA transcriptional levels of *TREM2* and *ARG1* being further subcategorized by IPI risk scores. **G** For M-MDSCs isolated from PB of the latest 31 DLBCL patients, the positive correlation between normalized MFI changes of surface TREM2 and intracellular ARG1 utilizing flow-cytometry analysis (Pearson’s *R* value = 0.738, *P* < 0.001). ΔΔCT, delta delta cycle threshold; ARG1, arginase 1; HD, healthy donor; IPI, international prognostic index; M-MDSCs, monocytic myeloid-derived suppressor cells; MFI, mean fluorescence intensity; mRNA, messenger ribonucleic acid; *n*, number; PB, peripheral blood; TREM2, triggering receptors expressed on myeloid cells 2

While the higher level of surface TREM2 on M-MDSCs predicted worse PFS and OS, our study also demonstrated that patients with lower absolute counts of CD8^+^ T cells (< 390/µL) in PB had significantly worse PFS and OS than those with higher absolute counts of CD8^+^ T cells in PB (Additional file 1: Figure S5Q, R). Therefore, we wonder if any association exists between the surface-TREM2 level on M-MDSCs and the absolute count of CD8^+^ T cells in PB. As shown in Fig. 4A, B, patients with higher normalized MFI change of surface TREM2 on M-MDSCs had significantly lower absolute count of CD8^+^ T cells in PB; in contrast, patients with lower normalized MFI change of surface TREM2 on M-MDSCs showed a significantly higher absolute count of CD8^+^ T cells in PB. Regarding the IPI risk score, the absolute counts of PB CD8^+^ T cells from high-risk DLBCL patients were significantly lower than those from low-risk DLBCL patients (Fig. 4C).

### Higher surface-TREM2 level on M-MDSCs correlated with higher intracellular ARG1 level within M-MDSCs

MDSCs had been found to secrete ARG1, causing L-arginine deprivation and T-cell proliferation arrest [31]. Additionally, both TREM2 and ARG1 are key hallmarks of M2-skewed macrophages [32, 33], and TREM2 regulates the expression of *ARG1* in macrophages [18]. Given that the surface-TREM2 level on M-MDSCs was seen as negatively associated with the absolute count of CD8^+^ T cells in PB, we hypothesize that the level of surface TREM2 and intracellular ARG1 are positively correlated in M-MDSCs. To prove the hypothesis, the mRNA transcriptional levels (ΔΔCT) of *TREM2* and *ARG1* in PBWCs were firstly analyzed, and we noticed that the mRNA transcriptional levels of *TREM2* and *ARG1* in PBWCs show a strong correlation (Fig. 4D; Additional file 1: Figure S1C, D). Of note, the mRNA transcriptional levels of both *TREM2* and *ARG1* in PBWCs were significantly higher in patients with higher IPI scores (Fig. 4E, F; Additional file 1: Figure S1C, D).

Because PBWCs comprised heterogenous cells, circulating M-MDSCs derived from the latest 31 DLBCL patients were analyzed for the relationship between intracellular ARG1 and surface TREM2 of M-MDSCs via flow-cytometry analysis. As shown in Fig. 4G, the level of intracellular ARG1 is positively correlated with the level of surface TREM2.

### Trem2 impaired proliferation of CD8^+^ T cells by Arg1 in *in-vitro* MDSC system

**Fig. 5:**
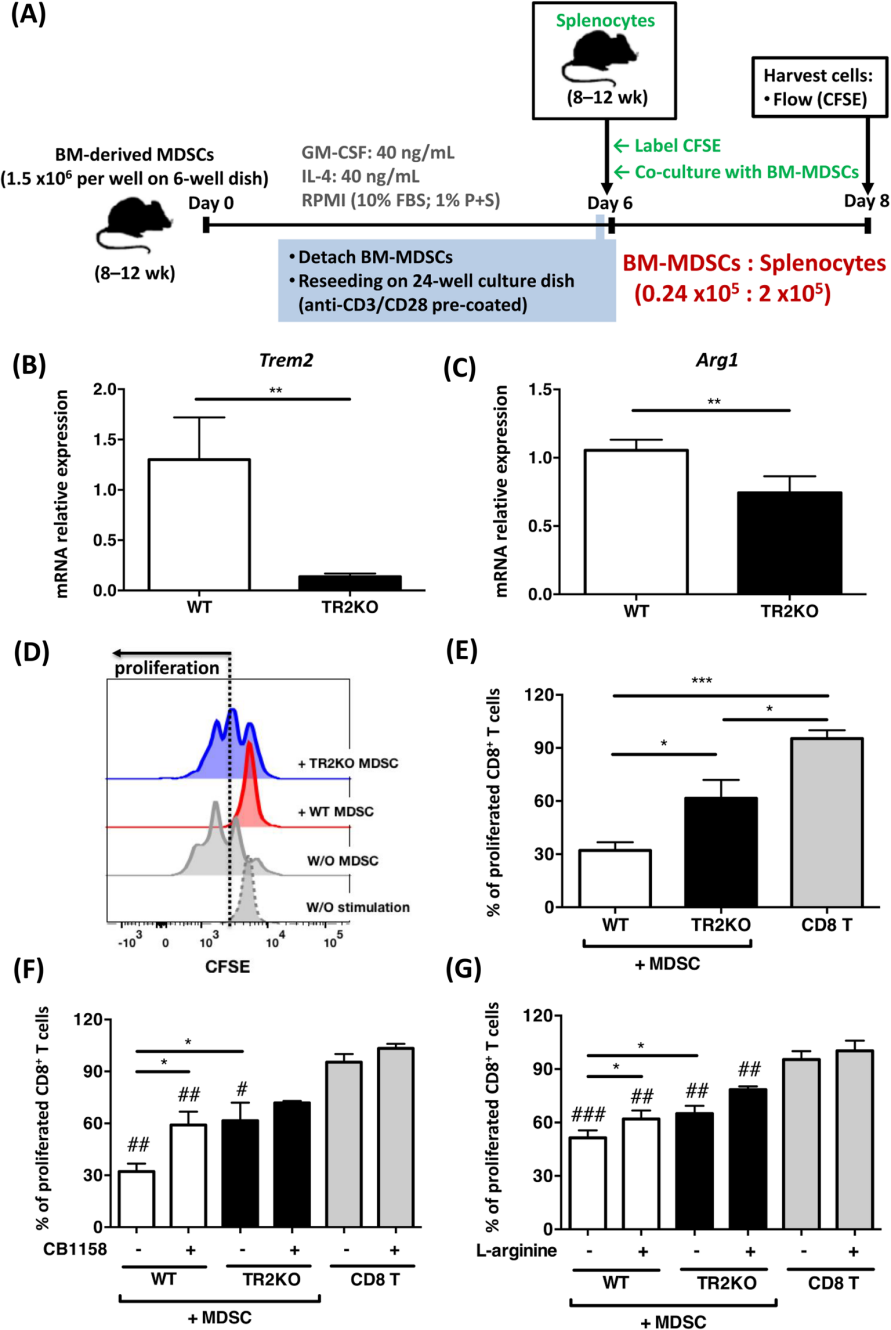
Induction of arginase 1 for MDSC-mediated CD8^+^ T-cell suppression being Trem2 dependent. **A** Schema of study design. **B**, **C** mRNA transcriptional level of *Trem2* and *Arg1* in BM-MDSCs from WT or *Trem2* KO mice; data being presented as fold change relative to the GAPDH mRNA transcriptional level. **D**–**G** CFSE-labeled splenocytes from naïve mice being co-cultured with 12% of WT or *Trem2* KO BM-MDSCs in the presence of L-arginine or CB-1158 (inhibitor of Arg1) for an additional two days; proliferation being assessed by flow cytometry, and histograms of proliferated population being gated on CD8^+^ T cells; data from two representatives of three reproducible experiments; Mean ± SEM of each group being presented in results (*n* = 6); for comparisons between two subgroups, differences being evaluated by Mann–Whitney nonparametric test: * (*P* < 0.05), ** (*P* < 0.01), or *** (*P* < 0.001); when comparing to the sole CD8.^+^ T subgroup (without MDSCs, CB1158, and L-arginine), differences being demonstrated as # (*P* < 0.05), ## (*P* < 0.01), or ### (*P* < 0.001) according to the Mann–Whitney nonparametric test. Arg1, arginase 1; BM, bone marrow; MDSCs, myeloid-derived suppressor cells; WT, wild-type; TR2KO, *Trem2* knockout; *Trem2*, triggering receptors expressed on myeloid cells 2

Given that the level of surface TREM2 on M-MDSCs was positively correlated to intracellular ARG1 and negatively associated with the absolute count of PB CD8^+^ T cells in DLBCL patients, we hypothesize that TREM2 may regulate the expression of ARG1 to impair the proliferation of CD8^+^ T cells. To validate the causal relationship between TREM2 on M-MDSCs cell surface, ARG1 from M-MDSCs, and CD8^+^ T cells, we adopted an in-vitro MDSCs system (Fig. 5A) [29, 30] by utilizing primary BM cells derived from B6 mice under the consideration that (1) *Trem2* KO mice were constructed on the B6 mouse strain and (2) currently there are no commercially available B-cell lymphoma cell lines that originate from B6 mice.

As shown in Fig. 5B, C, in-vitro MDSCs from *Trem2* KO mice demonstrated a significantly lower mRNA transcriptional level of *Arg1* than that from wild-type mice. Additionally, *Trem2* KO MDSCs showed a significantly weaker ability to suppress the proliferation of co-cultured CD8^+^ T cells than wild-type MDSCs (Fig. 5, D and E). Furthermore, the ability of wild-type MDSCs to suppress the proliferation of CD8^+^ T cells could be attenuated by supplementing additional L-arginine (Fig. 5G) or adding an Arg1 inhibitor (CB-1588) (Fig. 5F).

## Discussion

Contemporary immunotherapy is based mostly on activating or manipulating the functions of CTLs, including ICI targeting on PD-1, bispecific T-cell engagers (BiTEs), and CART therapy. CTLs play as the key effector cells in cancer immunity, and our study demonstrates that circulating CTLs matter in the prognosis of DLBCL patients, as patients with lower-IPI risk have significantly higher absolute counts of CTLs in PB (Fig. 4C), and such a finding could partially explain how patients with lower-IPI risk recur less (Additional file 1: Figure S5E, F).

Additionally, circulating M-MDSCs have been recently recognized as a poor prognostic factor for newly diagnosed DLBCL and as one of mechanisms causing resistance to CART therapy for relapsed or refractory DLBCL [10, 15, 17]. Our study confirms that DLBCL patients with higher IPI-risk scores have significantly higher percentages of circulating M-MDSCs among PBWCs (Fig. 1B), and patients with higher percentages of circulating M-MDSCs among PBWCs suffer from significantly worse PFS and OS (Fig. 1C, D).

More importantly, our human study has discovered that the level of surface TREM2 on circulating M-MDSCs is negatively associated with the absolute count of circulating CTLs (Fig. 4A). For the murine *in-vitro* MDSC system, we prove that *Trem2* KO MDSCs have attenuated ability to inhibit the proliferation of CTLs (Fig. 5D, E). The above two findings support the critical role of TREM2 in modulating immunity against cancer. A recent study using a murine fibrosarcoma model showed that the tumor on *Trem2* KO mice grew much slower than the tumor on WT mice [34], consistent with our original hypothesis that the expression of TREM2 on immunosuppressive myeloid-origin cells could facilitate the growth of tumors.

Our study further reveals the positive correlation between surface TREM2 and intracellular ARG1 of M-MDSCs (Fig. 4G). According to the mRNA transcriptional levels of PBWCs, flow-cytometry analysis of M-MDSCs, and murine in-vitro MDSC system, our study proves that ARG1 could be an important effector molecule downstream of the TREM2 signaling to affect the proliferation of CTLs. By utilizing integrated technology coupling single-cell RNA sequencing and intracellular protein activity in a murine cancer model, Katzenelenbogen also clearly demonstrated the highly correlated relationship between Trem2 and Arg1 [34]. In fact, depleting microenvironmental arginine through ARG1 is one of the first T-cell suppressive mechanisms described in MDSCs, and such a mechanism is mostly implicated in PMN-MDSCs [31, 35], but our study indicates that M-MDSCs can also be an important source for producing ARG1 in a lymphoma microenvironment.

The interplay between tumor microenvironments and lymphoma cells can be subcategorized into three major patterns: (1) recruitment, (2) re-education, and (3) effacement [36]. Regarding aggressive NHL cells, they lean toward inducing the effacement of the tumor microenvironment because these aggressive lymphoma cells proliferate rapidly, causing overwhelming destruction of the immune system within the tumor microenvironment, and leading to the paucity of reactive CD8^+^ T cells. Our results are highly consistent with the above theory. DLBCL patients with a higher-risk IPI show a significantly higher surface-TREM2 level on M-MDSCs (Fig. 2B) and a significantly lower absolute count of CD8^+^ T cells in PB (Fig. 4C), implicating that high-risk DLBCL causes devastating reduction in the number of circulating CD8^+^ T cells by TREM2-associated signaling.

The ratio of PB absolute lymphocyte count to absolute monocyte count (LMR) at diagnosis has been proposed to predict prognosis in patients with lymphoma, as patients with a lower LMR have demonstrated lower complete remission rates, PFS, and OS [37–39]. Interestingly, our study elucidates the key rationale behind LMR in predicting the prognosis of patients with lymphoma. According to our findings, both a lower absolute count of circulating CTLs (Additional file 1: Figure S5Q, R) and a higher percentage of circulating M-MDSCs among PBWCs (Fig. 1C, D) are associated with poor survival in DLBCL patients. Therefore, LMR could actually represent a simplified surrogate marker of the ratio of circulating CTLs to M-MDSCs. In other words, when predicting the prognosis of DLBCL patients, CTLs and M-MDSCs in PB could be much more precise than the absolute lymphocyte count and absolute monocyte count, respectively.

Our study has several limitations. First, occasional discrepancies could still exist between clinical outcomes and surface-TREM2 levels on M-MDSCs, and we must emphasize that surface TREM2 on circulating M-MDSCs is not a substitute for current risk-stratification systems, such as IPI scores. Yet as a novel prognostic factor, it provides important additional information with proper respect to the clinical context. Second, we did not adopt M-MDSCs from a murine lymphoma model because of lacking suitable B-cell lymphoma cell lines originating from the B6 mouse strain, which we are currently establishing. Third, our study is incapable of answering how these circulating M-MDSCs from DLBCL patients express a higher surface TREM2, as upstream signals originating from the tumor site must play the key tune, and this warrants further investigation by bioinformatics analyses [40].

## Conclusions

In summary, for newly diagnosed and treatment-naïve DLBCL adults, a higher level of surface TREM2 on circulating M-MDSCs is a poor prognostic factor for both PFS and OS, positively correlated with a higher level of intracellular ARG1 of M-MDSCs, and negatively associated with a lower absolute count of CD8^+^ T cells in PB. Surface TREM2 on M-MDSCs warrants further investigation for validating its possibility as a novel target in immunotherapy against DLBCL.

## Supplementary Information

**Additional file 1: Additional Figures, Figures S1–S6. Figure S1.** Healthy donors. Histogram of (A) surface-TREM2 level on M-MDSCs and (B) intracellular ARG1 level within M-MDSCs; red line denoting the healthy control for flow-cytometry analysis of surface TREM2 and intracellular ARG1. The mRNA transcriptional level of (C) *TREM2* and (D) *ARG1* being calculated using ΔCT. The absolute counts of (E) CD4^+^ T cells and (F) CD8^+^ T cells in PB. ΔCT, delta cycle threshold; ARG1, arginase 1; F, female; M, male; M-MDSCs, monocytic myeloid-derived suppressor cells; mRNA, messenger ribonucleic acid; *n*, number; PB, peripheral blood; TREM2, triggering receptors expressed on myeloid cells 2. **Figure S2.** Enrollment algorithm for newly diagnosed and treatment-naïve DLBCL patients. CNS, central nervous system; HIV, human immunodeficiency virus. **Figure S3.** Progression-free and overall survival for all 100 DLBCL patients. DLBCL, diffuse large B-cell lymphoma; *n*, number; OS, overall survival; PFS, progression-free survival. **Figure S4.** Outcomes by treatment-related factors. Progression-free and overall survival being subcategorized by frontline treatment (A, B), induction regimen (C, D), or treatment response after frontline treatment (E, F); *P* as the log-rank test for Kaplan–Meier estimate. CR, complete remission; mOS, median overall survival; mPFS, median progression-free survival; *n*, number; NR, non-reach; PD, progressive disease; PR, partial remission; SD, stable disease. **Figure S5.** Prognostic factors of DLBCL patients. Progression-free and overall survival being subcategorized by (A, B) age, (C, D) sex, (E, F) IPI-risk score, (G, H) bulky mass, (I, J) bone marrow involvement, (K, L) cell of origin, (M, N) double-expressor lymphoma, (O, P) absolute count of CD4^+^ T cells in PB, or (Q, R) absolute count of CD8^+^ T cells in PB; the cut-off values of age, absolute counts of CD4^+^ T cells and CD8^+^ T cells in PB determined by their medians; *P* as the log-rank test for Kaplan–Meier estimate. DEL, double-expressor lymphoma; GCB, germinal center B cell; IPI, international prognostic index; mOS, median overall survival; mPFS, median progression-free survival; *n*, number; NR, non-reach; PB, peripheral blood. **Figure S6.** Subgroup analysis of hazard ratios for (A) progression-free and (B) overall survival. BM, bone marrow; CI, confidence interval; GCB, germinal center B cell; HR, hazard ratio; IPI, international prognostic index; M-MDSCs, monocytic myeloid-derived suppressor cells; MFI, mean fluorescence intensity; OS, overall survival; PB, peripheral blood; PFS, progression-free survival; Pt. no., patient number; TREM2, triggering receptors expressed on myeloid cells 2.

## Data Availability

The datasets analyzed during the study are available on reasonable request.

## Abbreviations

ΔΔCT: Delta delta cycle threshold
ALCs: Absolute lymphocyte counts
ARG1: Arginase 1
BiTEs: Bispecific T-cell engagers
BM: Bone marrow
CARTs: Chimeric antigen receptor T-cells
CI: Confidence interval
CNS: Central nervous system
COO: Cell of origin
CR: Complete remission
CTLs: Cytotoxic T lymphocytes
DEL: Double-expressor lymphoma
DLBCL: Diffuse large B-cell lymphoma
F: Female
GCB: Germinal center B cell
HC: Healthy control
HD: Healthy donor
HIV: Human immunodeficiency virus
HR: Hazard ratio
ICIs: Immune checkpoint inhibitors
IHC: Immunohistochemistry
IPI: International prognostic index
M: Male
M-MDSCs: Monocytic myeloid-derived suppressor cells
MDSCs: Myeloid-derived suppressor cells
MFI: Mean fluorescence intensity
mOS: Median overall survival
mPFS: Median progression-free survival
mRNA: Messenger ribonucleic acid
n: Number
NR: Non-reach
OS: Overall survival
PB: Peripheral blood
PBWCs: Peripheral-blood white cells
PD: Progressive disease
PET: Positron emission tomography
PFS: Progression-free survival
PMN-MDSCs: Polymorphonucler myeloid-derived suppressor cells
PR: Partial remission
Pt. no.: Patient numbers
SD: Stable disease
TR2KO: *Trem2* Knockout
TREM2: Triggering receptors expressed on myeloid cells 2
WT: Wild-type
