# Supplementary material for: Surface TREM2 on circulating M-MDSCs as a novel prognostic factor for adults with treatment-naïve diffuse large B-cell lymphoma
Source: Exp Hematol Oncol. 2023 Apr 7;12:35. doi: 10.1186/s40164-023-00399-x (PMC10080769; doi:10.1186/s40164-023-00399-x)
Supplement: Supplementary file 1 — Additional file 1: Additional Figures, Figures S1–S6. Figure S1. Healthy donors. Histogram of (A) surface-TREM2 level on M-MDSCs and (B) intracellular ARG1 level within M-MDSCs; red line denoting the healthy control for flow-cytometry analysis of surface TREM2 and intracellular ARG1. The mRNA transcriptional level of (C) TREM2 and (D) ARG1 being calculated using ΔCT. The absolute counts of (E) CD4+ T cells and (F) CD8+ T cells in PB. ΔCT, delta cycle threshold; ARG1, arginase 1; F, female; M, male; M-MDSCs, monocytic myeloid-derived suppressor cells; mRNA, messenger ribonucleic acid; n, number; PB, peripheral blood; TREM2, triggering receptors expressed on myeloid cells 2. Figure S2. Enrollment algorithm for newly diagnosed and treatment-naïve DLBCL patients. CNS, central nervous system; HIV, human immunodeficiency virus. Figure S3. Progression-free and overall survival for all 100 DLBCL patients. DLBCL, diffuse large B-cell lymphoma; n, number; OS, overall survival; PFS, progression-free survival. Figure S4. Outcomes by treatment-related factors. Progression-free and overall survival being subcategorized by frontline treatment (A, B), induction regimen (C, D), or treatment response after frontline treatment (E, F); P as the log-rank test for Kaplan–Meier estimate. CR, complete remission; mOS, median overall survival; mPFS, median progression-free survival; n, number; NR, non-reach; PD, progressive disease; PR, partial remission; SD, stable disease. Figure S5. Prognostic factors of DLBCL patients. Progression-free and overall survival being subcategorized by (A, B) age, (C, D) sex, (E, F) IPI-risk score, (G, H) bulky mass, (I, J) bone marrow involvement, (K, L) cell of origin, (M, N) double-expressor lymphoma, (O, P) absolute count of CD4+ T cells in PB, or (Q, R) absolute count of CD8+ T cells in PB; the cut-off values of age, absolute counts of CD4+ T cells and CD8+ T cells in PB determined by their medians; P as the log-rank test for Kaplan–Meier estimate. DEL, d [file 40164_2023_399_MOESM1_ESM.pdf]

Supplementary Data

Surface TREM2 on circulating M-MDSCs as a novel prognostic factor for adults with treatment-naïve diffuse large B-cell lymphoma

Running title: Surface TREM2 on M-MDSCs in DLBCL

Hao-Yuan Wang,[1,2,3] Fu-Chen Yang,[4] Ching-Fen Yang,[5] Yao-Chung Liu,[1,2] Po-Shen Ko,[1,2] Chien-Jung Li,[4] Chun-Kuang Tsai,[1,2] Yi-Lin Chung,[6] Nien-Jung Chen[2,4]

[1] Division of Hematology and Oncology, Department of Medicine, Taipei Veterans General Hospital, Taipei, Taiwan (R.O.C.)

[2] Faculty of Medicine, School of Medicine, National Yang Ming Chiao Tung University, Taipei, Taiwan (R.O.C.)

[3] Program in Molecular Medicine, School of Life Sciences, National Yang Ming Chiao Tung University, Taipei, Taiwan (R.O.C.)

[4] Institute of Microbiology and Immunology, School of Life Sciences, National Yang Ming Chiao Tung University, Taipei, Taiwan (R.O.C.)

[5] Department of Pathology and Laboratory Medicine, Taipei Veterans General Hospital, Taipei, Taiwan (R.O.C.)

[6] Institute of Genome Sciences, School of Life Sciences, National Yang Ming Chiao Tung University, Taipei, Taiwan (R.O.C.)

Legends for supplementary digital content		Page
**Figure S1**	**Healthy donors.** Histogram of **(A)** surface-TREM2 level on M-MDSCs and **(B)** intracellular ARG1 level within M-MDSCs; red line denoting the healthy control for flow-cytometry analysis of surface TREM2 and intracellular ARG1. The mRNA transcriptional level of **(C)** *TREM2* and **(D)** *ARG1* being calculated using ΔCT. The absolute counts of **(E)** $CD4^+$ T cells and **(F)** $CD8^+$ T cells in PB.<br><br>△CT, delta cycle threshold; ARG1, arginase 1; F, female; M, male; M-MDSCs, monocytic myeloid-derived suppressor cells; mRNA, messenger ribonucleic acid; *n*, number; PB, peripheral blood; TREM2, triggering receptors expressed on myeloid cells 2	**3–4**
**Figure S2**	**Enrollment algorithm for newly diagnosed and treatment-naïve DLBCL patients.**<br><br>CNS, central nervous system; HIV, human immunodeficiency virus	**5**
**Figure S3**	**Progression-free and overall survival for all 100 DLBCL patients.**<br><br>DLBCL, diffuse large B-cell lymphoma; *n*, number; OS, overall survival; PFS, progression-free survival	**6**
**Figure S4**	**Outcomes by treatment-related factors.** Progression-free and overall survival being subcategorized by frontline treatment **(A, B)**, induction regimen **(C, D)**, or treatment response after frontline treatment **(E, F)**; *P* as the log-rank test for Kaplan–Meier estimate.<br><br>CR, complete remission; mOS, median overall survival; mPFS, median progression-free survival; *n*, number; NR, non-reach; PD, progressive disease; PR, partial remission; SD, stable disease	**7–8**
**Figure S5**	**Prognostic factors of DLBCL patients.** Progression-free and overall survival being subcategorized by **(A, B)** age, **(C, D)** sex, **(E, F)** IPI-risk score, **(G, H)** bulky mass, **(I, J)** bone marrow involvement, **(K, L)** cell of origin, **(M, N)** double-expressor lymphoma, **(O, P)** absolute count of $CD4^+$ T cells in PB, or **(Q, R)** absolute count of $CD8^+$ T cells in PB.<br><br>DEL, double-expressor lymphoma; GCB, germinal center B cell; IPI, international prognostic index; mOS, median overall survival; mPFS, median progression-free survival; *n*, number; NR, non-reach; PB, peripheral blood	**9–12**
**Figure S6**	**Subgroup analysis of hazard ratios for (A) progression-free and (B) overall survival.**<br><br>BM, bone marrow; CI, confidence interval; GCB, germinal center B cell; HR, hazard ratio; IPI, international prognostic index; M-MDSCs, monocytic myeloid-derived suppressor cells; MFI, mean fluorescence intensity; OS, overall survival; PB, peripheral blood; PFS, progression-free survival; Pt. no., patient number; TREM2, triggering receptors expressed on myeloid cells 2	**13–15**

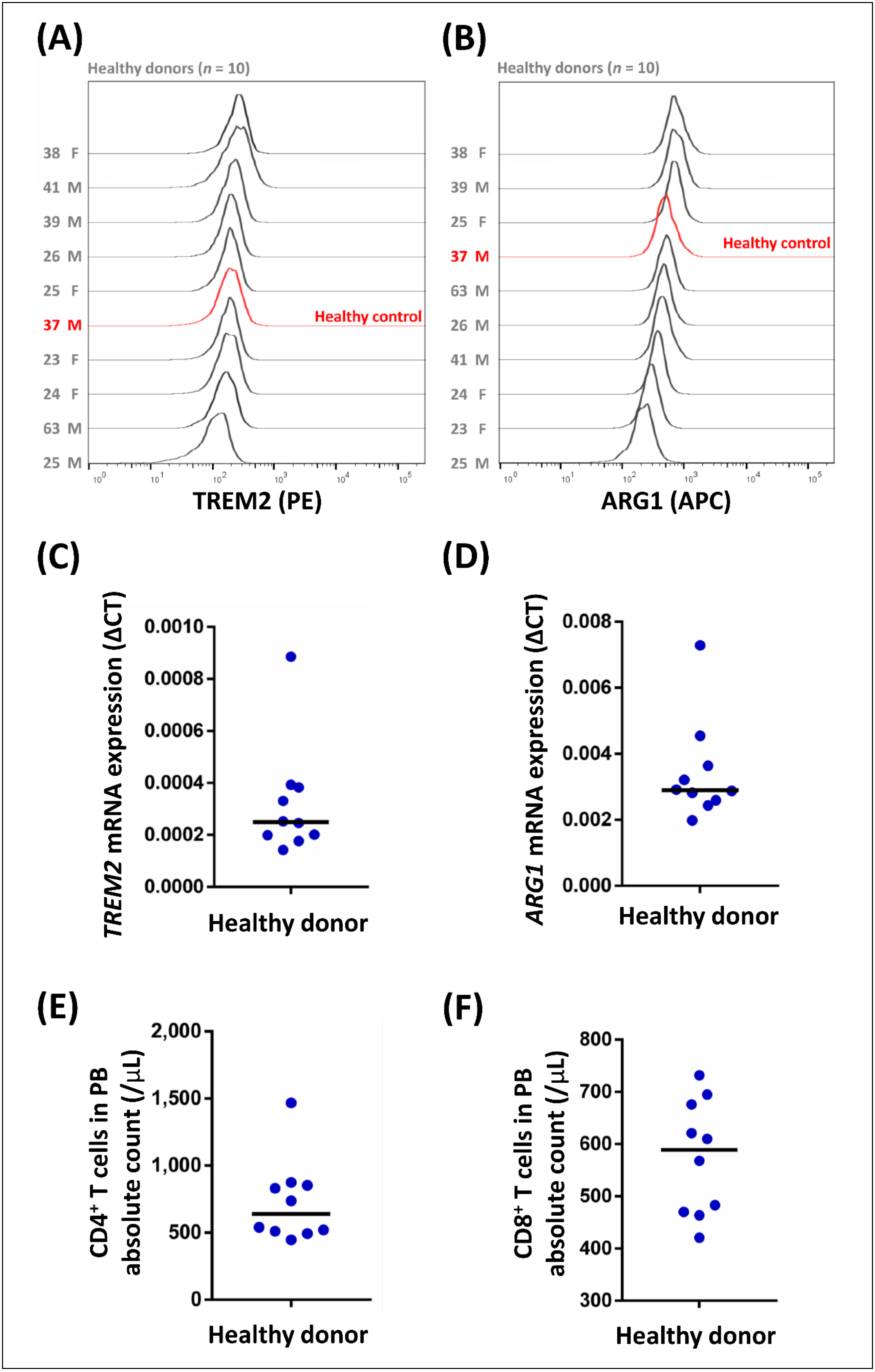

(A)
Healthy donors (n = 10)
38 F
41 M
39 M
26 M
25 F
37 M
23 F
24 F
63 M
25 M
Healthy control
TREM2 (PE)
(B)
Healthy donors (n = 10)
38 F
39 M
25 F
37 M
63 M
26 M
41 M
24 F
23 F
25 M
Healthy control
ARG1 (APC)
(C)
TREM2 mRNA expression (ΔCT)
0.0010
0.0008
0.0006
0.0004
0.0002
0.0000
Healthy donor
(D)
ARG1 mRNA expression (ΔCT)
0.008
0.006
0.004
0.002
0.000
Healthy donor
(E)
CD4+ T cells in PB absolute count (/μL)
2,000
1,500
1,000
500
0
Healthy donor
(F)
CD8+ T cells in PB absolute count (/μL)
800
700
600
500
400
300
Healthy donor

Figure S1. Healthy donors. Histogram of **(A)** surface-TREM2 level on M-MDSCs and **(B)** intracellular ARG1 level within M-MDSCs; red line denoting the healthy control for flow-cytometry analysis of surface TREM2 and intracellular ARG1. The mRNA transcriptional level of **(C)** *TREM2* and **(D)** *ARG1* being calculated using ΔCT. The absolute counts of **(E)** $CD4^+$ T cells and **(F)** $CD8^+$ T cells in PB.

△CT, delta cycle threshold; ARG1, arginase 1; F, female; M, male; M-MDSCs, monocytic myeloid-derived suppressor cells; mRNA, messenger ribonucleic acid; *n*, number; PB, peripheral blood; TREM2, triggering receptors expressed on myeloid cells 2

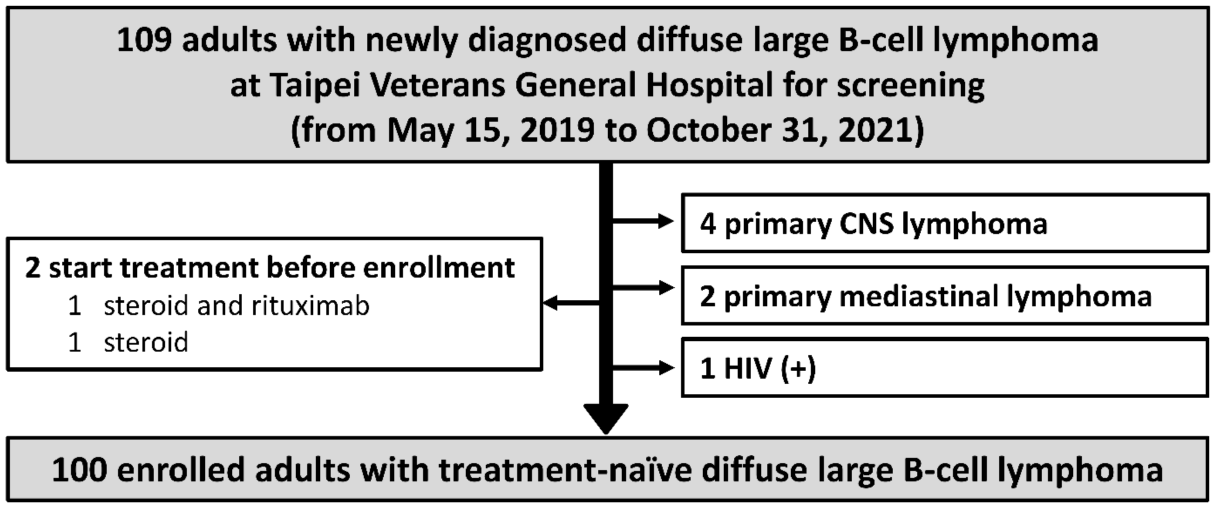

Figure S2. Enrollment algorithm for newly diagnosed and treatment-naïve DLBCL patients.

CNS, central nervous system; HIV, human immunodeficiency virus

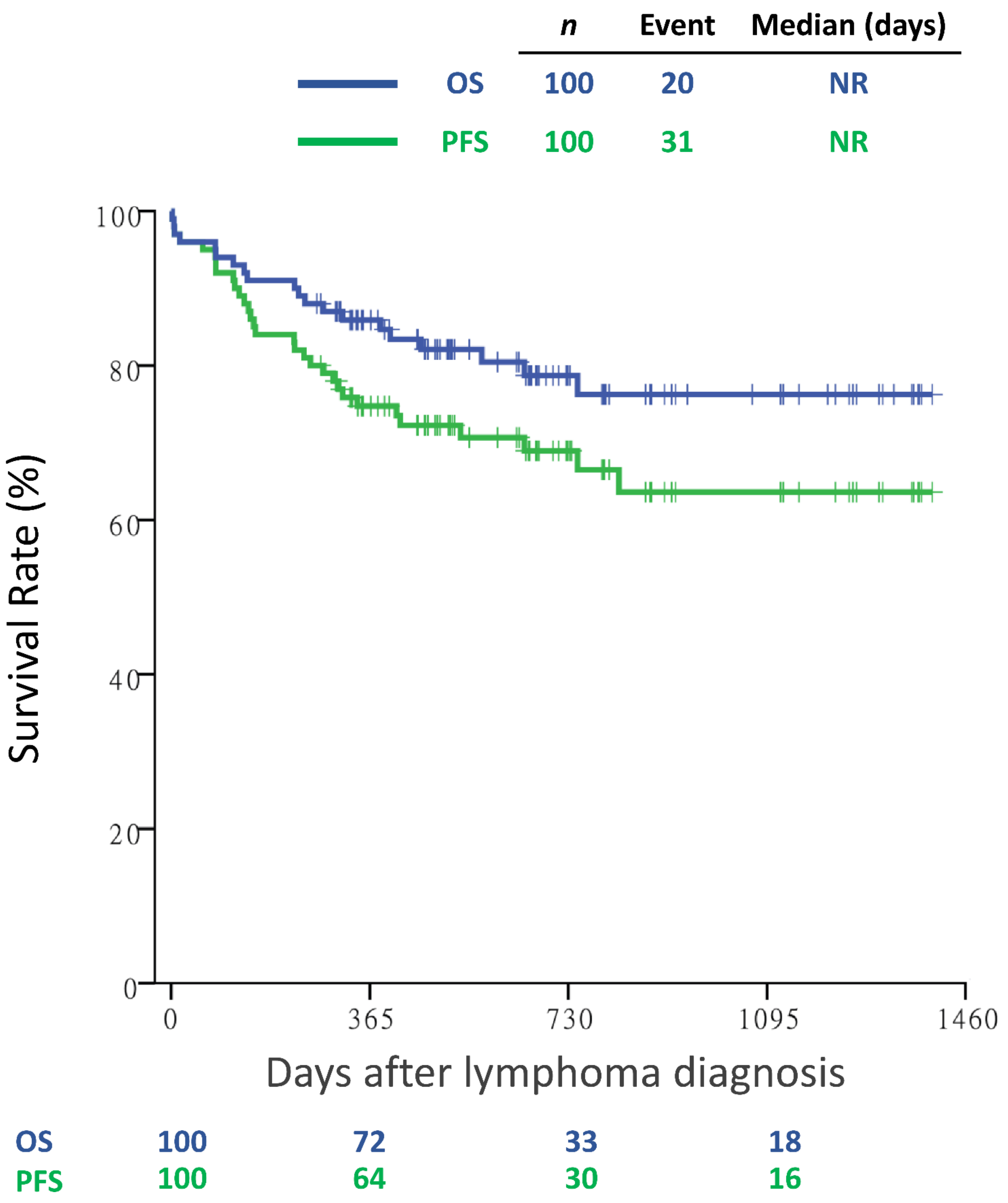

Figure S3. Progression-free and overall survival for all 100 DLBCL patients.

DLBCL, diffuse large B-cell lymphoma; *n*, number; OS, overall survival; PFS, progression-free survival

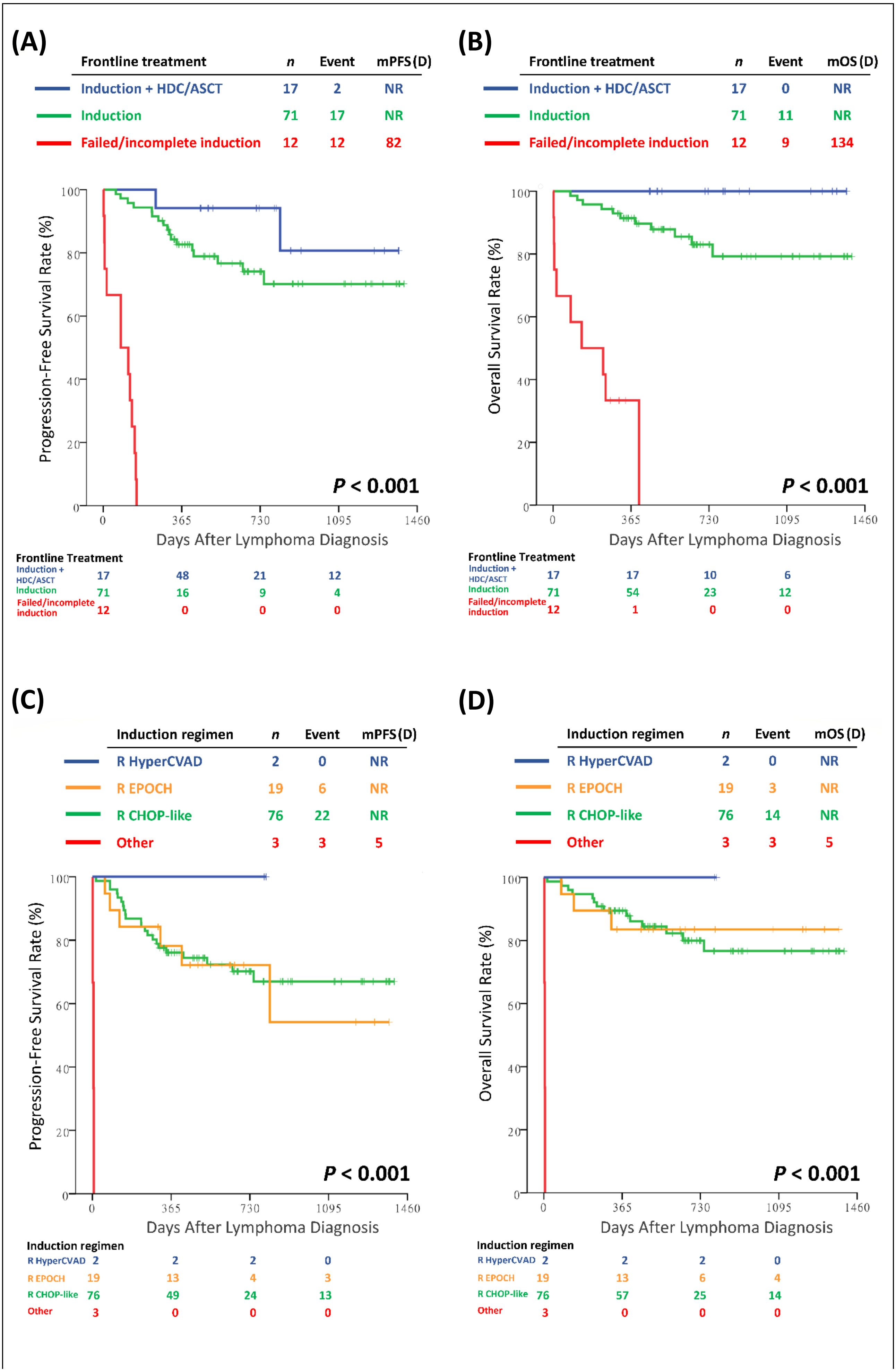
(A)
Frontline treatment | n | Event | mPFS (D)
Induction + HDC/ASCT | 17 | 2 | NR
Induction | 71 | 17 | NR
Failed/incomplete induction | 12 | 12 | 82
Progression-Free Survival Rate (%)
P < 0.001
Days After Lymphoma Diagnosis
0 365 730 1095 1460
Frontline Treatment
Induction + HDC/ASCT 17 48 21 12
Induction 71 16 9 4
Failed/incomplete induction 12 0 0 0
(B)
Frontline treatment | n | Event | mOS (D)
Induction + HDC/ASCT | 17 | 0 | NR
Induction | 71 | 11 | NR
Failed/incomplete induction | 12 | 9 | 134
Overall Survival Rate (%)
P < 0.001
Days After Lymphoma Diagnosis
0 365 730 1095 1460
Frontline Treatment
Induction + HDC/ASCT 17 17 10 6
Induction 71 54 23 12
Failed/incomplete induction 12 1 0 0
(C)
Induction regimen | n | Event | mPFS (D)
R HyperCVAD | 2 | 0 | NR
R EPOCH | 19 | 6 | NR
R CHOP-like | 76 | 22 | NR
Other | 3 | 3 | 5
Progression-Free Survival Rate (%)
P < 0.001
Days After Lymphoma Diagnosis
0 365 730 1095 1460
Induction regimen
R HyperCVAD 2 2 2 0
R EPOCH 19 13 4 3
R CHOP-like 76 49 24 13
Other 3 0 0 0
(D)
Induction regimen | n | Event | mOS (D)
R HyperCVAD | 2 | 0 | NR
R EPOCH | 19 | 3 | NR
R CHOP-like | 76 | 14 | NR
Other | 3 | 3 | 5
Overall Survival Rate (%)
P < 0.001
Days After Lymphoma Diagnosis
0 365 730 1095 1460
Induction regimen
R HyperCVAD 2 2 2 0
R EPOCH 19 13 6 4
R CHOP-like 76 57 25 14
Other 3 0 0 0

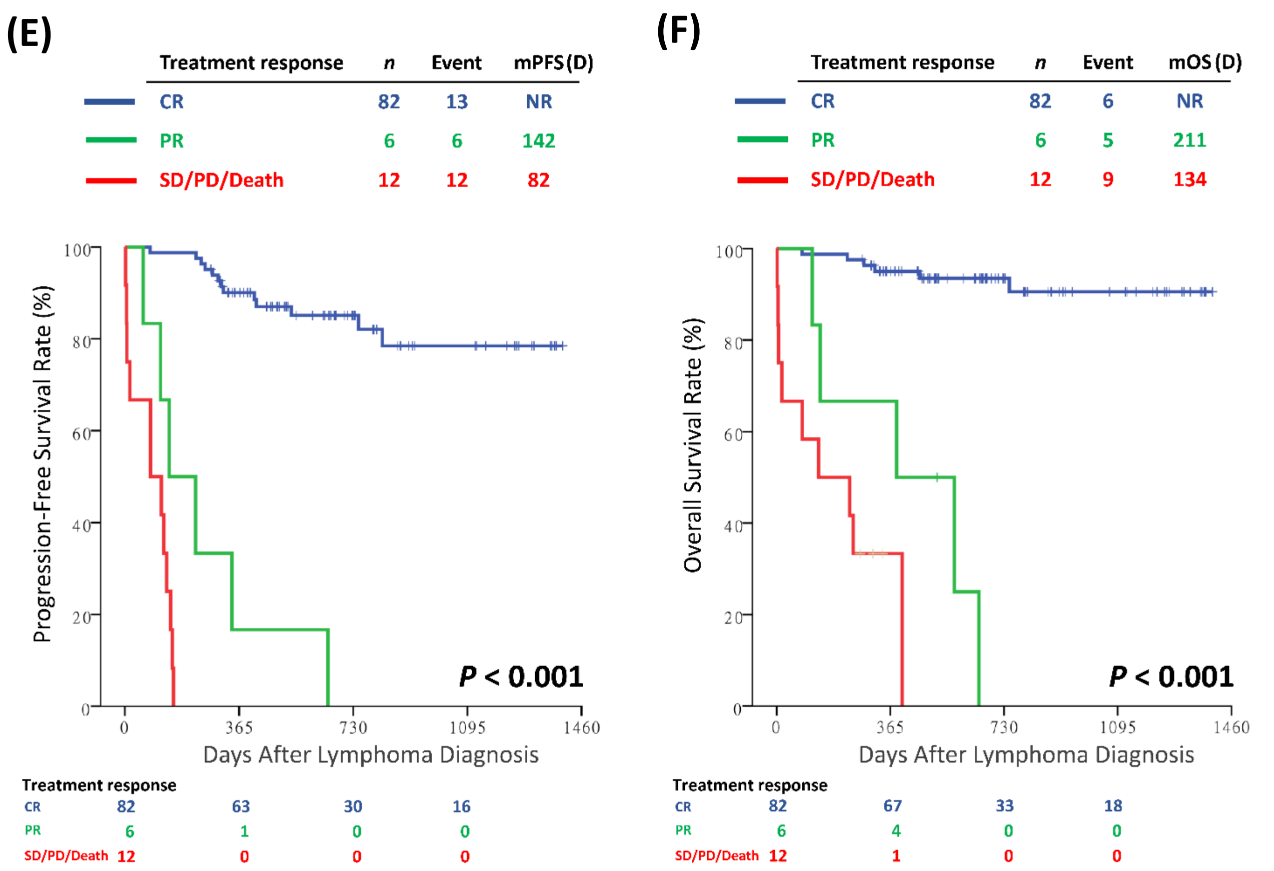

Figure S4. Outcomes by treatment-related factors. Progression-free and overall survival being subcategorized by frontline treatment **(A, B)**, induction regimen **(C, D)**, or treatment response after frontline treatment **(E, F)**; *P* as the log-rank test for Kaplan–Meier estimate.

CR, complete remission; mOS, median overall survival; mPFS, median progression-free survival; *n*, number; NR, non-reach; PD, progressive disease; PR, partial remission; SD, stable disease

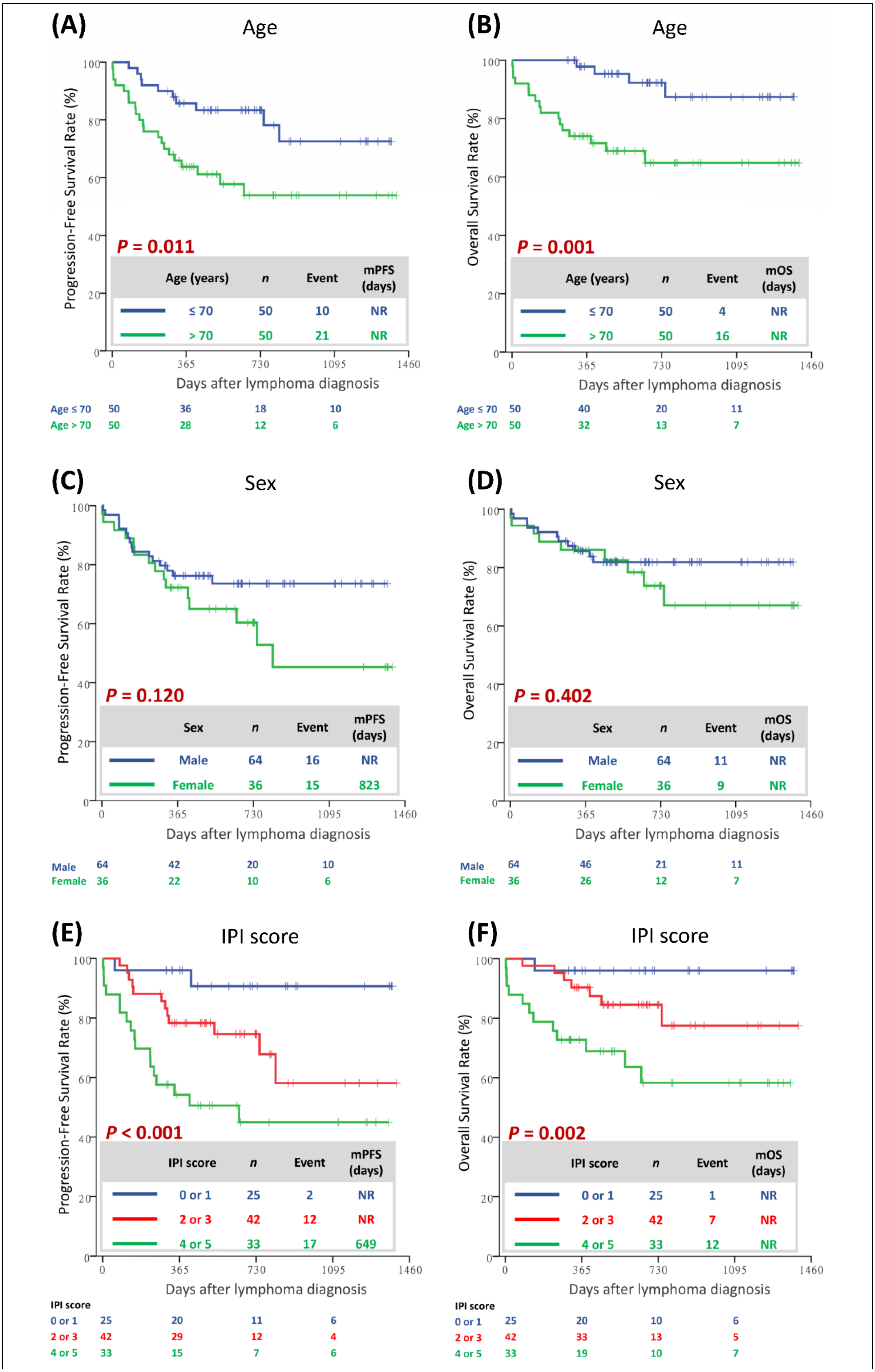
(A)
Age
Progression-Free Survival Rate (%)
P = 0.011
Age (years) n Event mPFS (days)
≤ 70 50 10 NR
> 70 50 21 NR
0 365 730 1095 1460
Days after lymphoma diagnosis
Age ≤ 70 50 36 18 10
Age > 70 50 28 12 6
(B)
Age
Overall Survival Rate (%)
P = 0.001
Age (years) n Event mOS (days)
≤ 70 50 4 NR
> 70 50 16 NR
0 365 730 1095 1460
Days after lymphoma diagnosis
Age ≤ 70 50 40 20 11
Age > 70 50 32 13 7
(C)
Sex
Progression-Free Survival Rate (%)
P = 0.120
Sex n Event mPFS (days)
Male 64 16 NR
Female 36 15 823
0 365 730 1095 1460
Days after lymphoma diagnosis
Male 64 42 20 10
Female 36 22 10 6
(D)
Sex
Overall Survival Rate (%)
P = 0.402
Sex n Event mOS (days)
Male 64 11 NR
Female 36 9 NR
0 365 730 1095 1460
Days after lymphoma diagnosis
Male 64 46 21 11
Female 36 26 12 7
(E)
IPI score
Progression-Free Survival Rate (%)
P < 0.001
IPI score n Event mPFS (days)
0 or 1 25 2 NR
2 or 3 42 12 NR
4 or 5 33 17 649
0 365 730 1095 1460
Days after lymphoma diagnosis
IPI score
0 or 1 25 20 11 6
2 or 3 42 29 12 4
4 or 5 33 15 7 6
(F)
IPI score
Overall Survival Rate (%)
P = 0.002
IPI score n Event mOS (days)
0 or 1 25 1 NR
2 or 3 42 7 NR
4 or 5 33 12 NR
0 365 730 1095 1460
Days after lymphoma diagnosis
IPI score
0 or 1 25 20 10 6
2 or 3 42 33 13 5
4 or 5 33 19 10 7

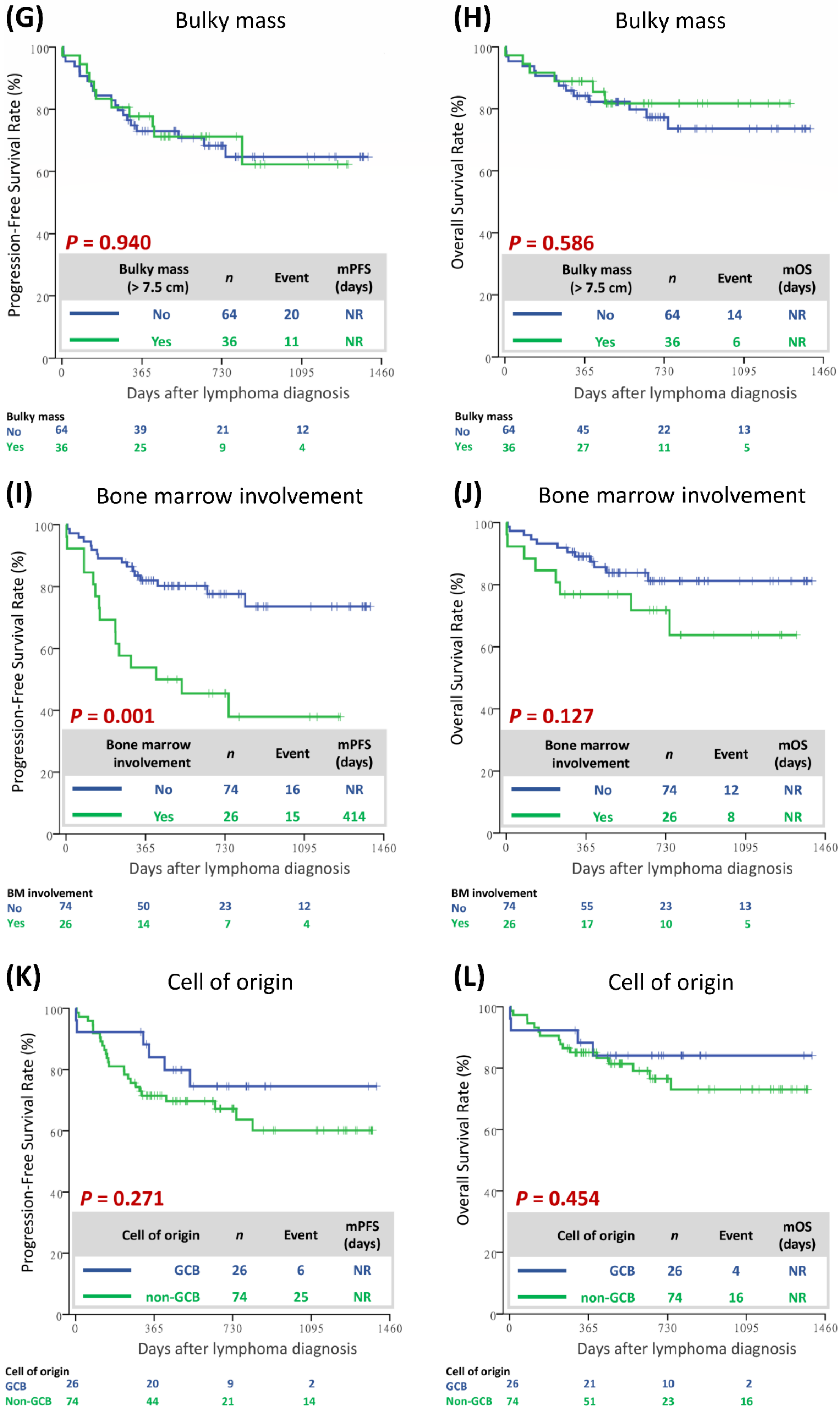
(G)
Bulky mass
Progression-Free Survival Rate (%)
100
80
60
40
20
0
P = 0.940
Bulky mass (> 7.5 cm) n Event mPFS (days)
No 64 20 NR
Yes 36 11 NR
0 365 730 1095 1460
Days after lymphoma diagnosis
Bulky mass
No 64 39 21 12
Yes 36 25 9 4
(H)
Bulky mass
Overall Survival Rate (%)
100
80
60
40
20
0
P = 0.586
Bulky mass (> 7.5 cm) n Event mOS (days)
No 64 14 NR
Yes 36 6 NR
0 365 730 1095 1460
Days after lymphoma diagnosis
Bulky mass
No 64 45 22 13
Yes 36 27 11 5
(I)
Bone marrow involvement
Progression-Free Survival Rate (%)
100
80
60
40
20
0
P = 0.001
Bone marrow involvement n Event mPFS (days)
No 74 16 NR
Yes 26 15 414
0 365 730 1095 1460
Days after lymphoma diagnosis
BM involvement
No 74 50 23 12
Yes 26 14 7 4
(J)
Bone marrow involvement
Overall Survival Rate (%)
100
80
60
40
20
0
P = 0.127
Bone marrow involvement n Event mOS (days)
No 74 12 NR
Yes 26 8 NR
0 365 730 1095 1460
Days after lymphoma diagnosis
BM involvement
No 74 55 23 13
Yes 26 17 10 5
(K)
Cell of origin
Progression-Free Survival Rate (%)
100
80
60
40
20
0
P = 0.271
Cell of origin n Event mPFS (days)
GCB 26 6 NR
non-GCB 74 25 NR
0 365 730 1095 1460
Days after lymphoma diagnosis
Cell of origin
GCB 26 20 9 2
Non-GCB 74 44 21 14
(L)
Cell of origin
Overall Survival Rate (%)
100
80
60
40
20
0
P = 0.454
Cell of origin n Event mOS (days)
GCB 26 4 NR
non-GCB 74 16 NR
0 365 730 1095 1460
Days after lymphoma diagnosis
Cell of origin
GCB 26 21 10 2
Non-GCB 74 51 23 16

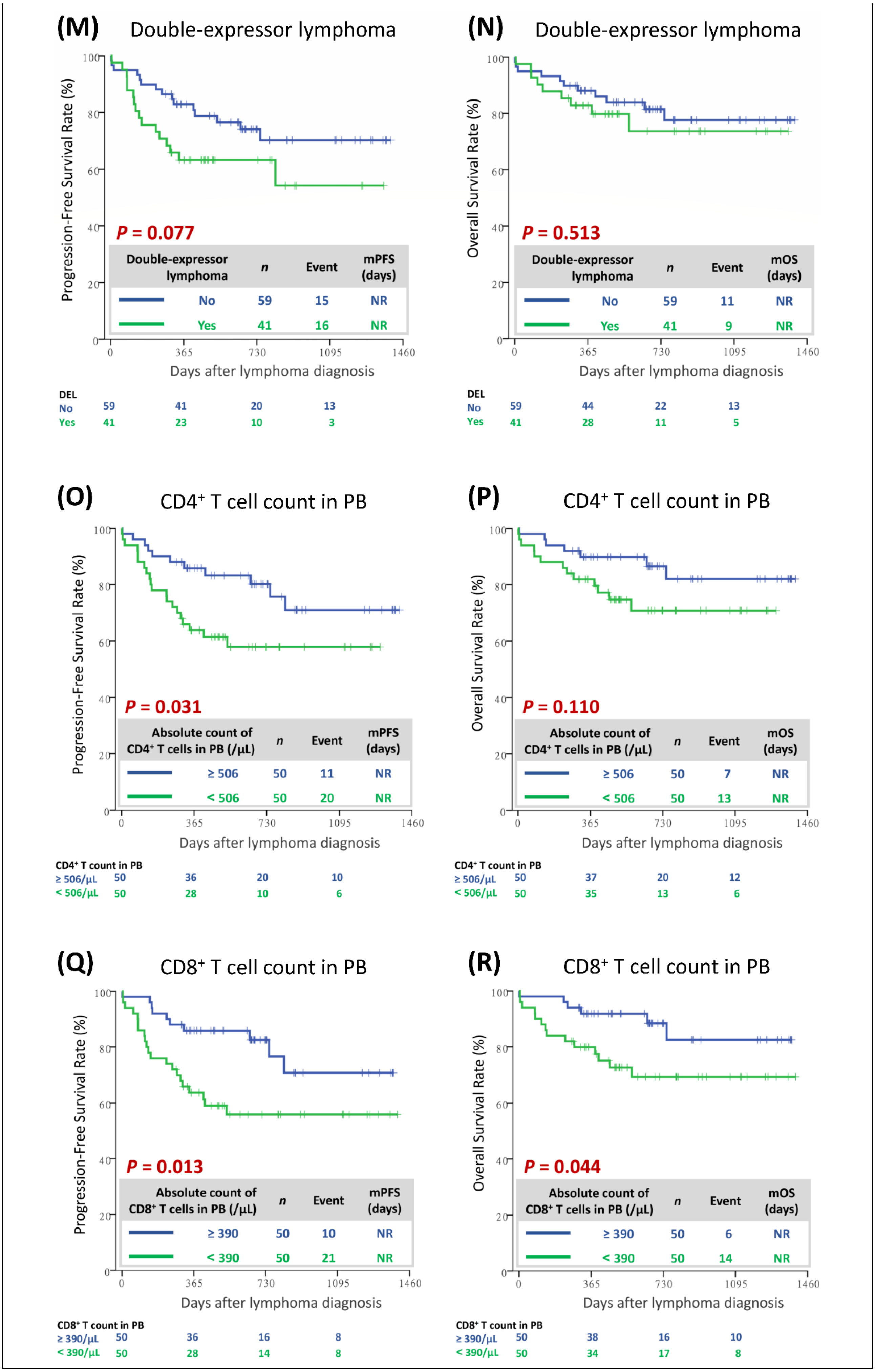

(M) Double-expressor lymphoma
Progression-Free Survival Rate (%)
P = 0.077
Double-expressor lymphoma | n | Event | mPFS (days)
No | 59 | 15 | NR
Yes | 41 | 16 | NR
Days after lymphoma diagnosis
0 365 730 1095 1460
DEL
No 59 41 20 13
Yes 41 23 10 3
(N) Double-expressor lymphoma
Overall Survival Rate (%)
P = 0.513
Double-expressor lymphoma | n | Event | mOS (days)
No | 59 | 11 | NR
Yes | 41 | 9 | NR
Days after lymphoma diagnosis
DEL
No 59 44 22 13
Yes 41 28 11 5
(O) CD4+ T cell count in PB
Progression-Free Survival Rate (%)
P = 0.031
Absolute count of CD4+ T cells in PB (/μL) | n | Event | mPFS (days)
≥ 506 | 50 | 11 | NR
< 506 | 50 | 20 | NR
Days after lymphoma diagnosis
CD4+ T count in PB
≥ 506/μL 50 36 20 10
< 506/μL 50 28 10 6
(P) CD4+ T cell count in PB
Overall Survival Rate (%)
P = 0.110
Absolute count of CD4+ T cells in PB (/μL) | n | Event | mOS (days)
≥ 506 | 50 | 7 | NR
< 506 | 50 | 13 | NR
Days after lymphoma diagnosis
CD4+ T count in PB
≥ 506/μL 50 37 20 12
< 506/μL 50 35 13 6
(Q) CD8+ T cell count in PB
Progression-Free Survival Rate (%)
P = 0.013
Absolute count of CD8+ T cells in PB (/μL) | n | Event | mPFS (days)
≥ 390 | 50 | 10 | NR
< 390 | 50 | 21 | NR
Days after lymphoma diagnosis
CD8+ T count in PB
≥ 390/μL 50 36 16 8
< 390/μL 50 28 14 8
(R) CD8+ T cell count in PB
Overall Survival Rate (%)
P = 0.044
Absolute count of CD8+ T cells in PB (/μL) | n | Event | mOS (days)
≥ 390 | 50 | 6 | NR
< 390 | 50 | 14 | NR
Days after lymphoma diagnosis
CD8+ T count in PB
≥ 390/μL 50 38 16 10
< 390/μL 50 34 17 8

Figure S5. Prognostic factors of DLBCL patients. Progression-free and overall survival being subcategorized by **(A, B)** age, **(C, D)** sex, **(E, F)** IPI-risk score, **(G, H)** bulky mass, **(I, J)** bone marrow involvement, **(K, L)** cell of origin, **(M, N)** double-expressor lymphoma, **(O, P)** absolute count of $CD4^+$ T cells in PB, or **(Q, R)** absolute count of $CD8^+$ T cells in PB; the cut-off values of age, absolute counts of $CD4^+$ T cells and $CD8^+$ T cells in PB determined by their medians; *P* as the log-rank test for Kaplan–Meier estimate.

DEL, double-expressor lymphoma; GCB, germinal center B cell; IPI, international prognostic index; mOS, median overall survival; mPFS, median progression-free survival; *n*, number; NR, non-reach; PB, peripheral blood

Subgroup	Pt. no.	Events	HR (95% CI)
Age (years)			
> 70	50	21	2.574 (1.210–5.473)
≦ 70	50	10	1
Sex			
Female	36	15	1.737 (0.858–3.516)
Male	64	16	1
IPI risk score (points)			
4 or 5	33	17	9.060 (2.089–39.300)
2 or 3	42	12	3.989 (0.891–17.849)
0 or 1	25	2	1
Bulky mass > 7.5 cm			
Yes	36	11	0.972 (0.466–2.031)
No	64	20	1
BM involvement			
Yes	26	15	3.238 (1.598–6.560)
No	74	16	1
Cell of origin (Hans algorithm)			
Non-GCB	74	25	1.642 (0.673–4.008)
GCB	26	6	1
Double expressor lymphoma			
Yes	41	16	1.874 (0.923–3.804)
No	59	15	1
Absolute count of $CD4^+$ T cells in PB (/μL)			
< 506	50	20	2.208 (1.054–4.624)
≧ 506	50	11	1
Absolute count of $CD8^+$ T cells in PB (/μL)			
< 390	50	21	2.507 (1.179–5.331)
≧ 390	50	10	1
Percentage of M-MDSCs among $CD45^+$ cells in PB (%)			
> 0.92	53	21	2.451 (1.147–5.234)
≦ 0.92	47	10	1
Normalized MFI change of surface TREM2 on M-MDSCs (%)			
> 44	26	15	10.681 (2.433–46.894)
2 to 44	48	14	4.822 (1.091–21.304)
< 2	26	2	1

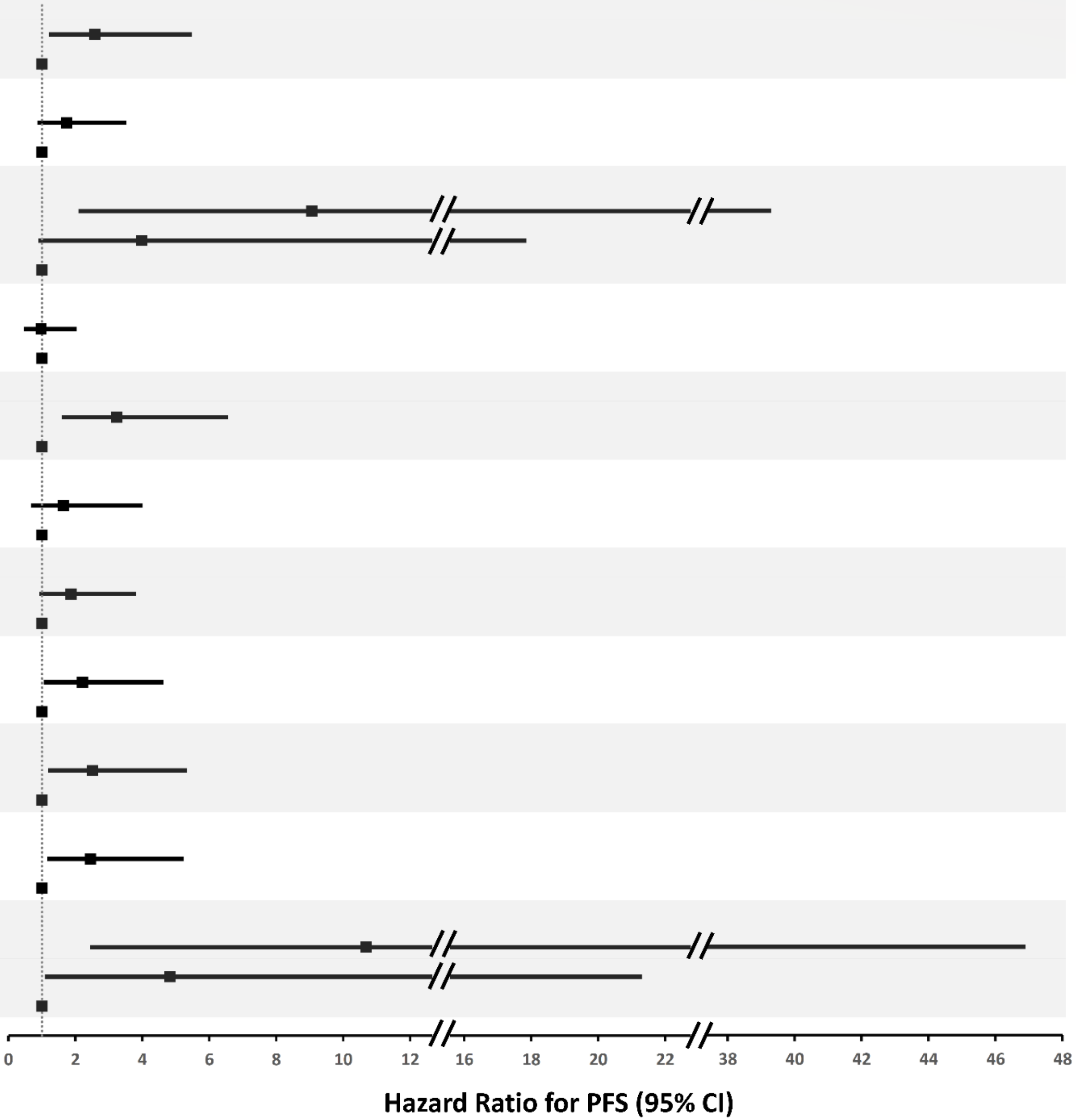

Subgroup	Pt. no.	Events	HR (95% CI)
Age (years)			
> 70	50	16	4.997 (1.667–14.975)
≦ 70	50	4	1
Sex			
Female	36	9	1.455 (0.603–3.512)
Male	64	11	1
IPI risk score (points)			
4 or 5	33	12	11.362 (1.476–87.444)
2 or 3	42	7	4.315 (0.531–35.088)
0 or 1	25	1	1
Bulky mass > 7.5 cm			
Yes	36	6	0.767 (0.295–1.998)
No	64	14	1
BM involvement			
Yes	26	8	1.981 (0.809–4.852)
No	74	12	1
Cell of origin (Hans algorithm)			
Non-GCB	74	16	1.516 (0.506–4.540)
GCB	26	4	1
Double expressor lymphoma			
Yes	41	9	1.342 (0.554–3.252)
No	59	11	1
Absolute count of $CD4^+$ T cells in PB (/μL)			
< 506	50	13	2.083 (0.829–5.233)
≧ 506	50	7	1
Absolute count of $CD8^+$ T cells in PB (/μL)			
< 390	50	14	2.577 (0.989–6.714)
≧ 390	50	6	1
Percentage of M-MDSCs among $CD45^+$ cells in PB (%)			
> 0.92	53	15	3.626 (1.303–10.093)
≦ 0.92	47	5	1
Normalized MFI change of surface TREM2 on M-MDSCs (%)			
> 44	26	11	13.690 (1.765–106.182)
2 to 44	48	8	5.014 (0.626–40.169)
< 2	26	1	1

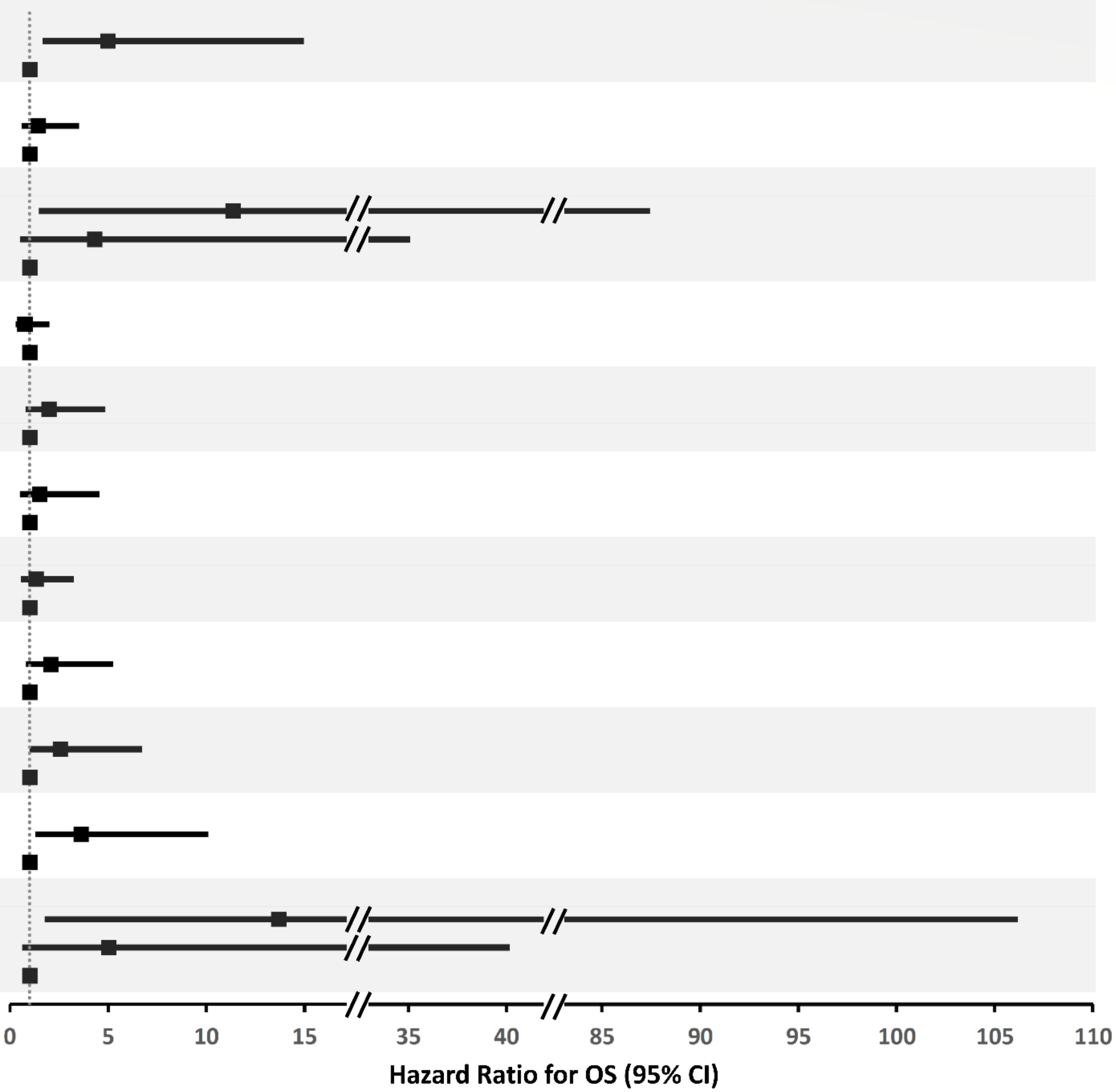

Figure S6. Subgroup analysis of hazard ratios for (A) progression-free and (B) overall survival.

BM, bone marrow; CI, confidence interval; GCB, germinal center B cell; HR, hazard ratio; IPI, international prognostic index; M-MDSCs, monocytic myeloid-derived suppressor cells; MFI, mean fluorescence intensity; OS, overall survival; PB, peripheral blood; PFS, progression-free survival; Pt. no., patient number; TREM2, triggering receptors expressed on myeloid cells 2
